# Decoding the Formation and Elimination Mechanism of Ethyl Carbamate in Strong-Aroma *Baijiu*

**DOI:** 10.3390/foods13233743

**Published:** 2024-11-22

**Authors:** Liqiang Zhang, Yue Qiu, Yongqing Zhang, Yintao Jia, Baoguo Sun, Wei Dong

**Affiliations:** 1 College of Food and Health, Beijing Technology and Business University, Beijing 100048, China; hellomervyn@126.com (L.Z.); sunbg@btbu.edu.cn (B.S.); 2 Key Laboratory of Brewing Molecular Engineering of China Light Industry, Beijing Technology and Business University, Beijing 100048, China; qiuyue010122@163.com (Y.Q.); yongqing2023@163.com (Y.Z.); jiayintao2022@163.com (Y.J.)

**Keywords:** ethyl carbamate, ethyl carbamate precursors, microbial community, fermentation and distillation process, strong-aroma *Baijiu*

## Abstract

In the present study, changes in the physicochemical indices, ethyl carbamate (EC) precursor and EC contents, and microbial communities of fermented grains under different fermentation patterns during strong-aroma *Baijiu* (SAB) fermentation and changes in EC precursor and EC contents during distillation were investigated to study EC formation during these processes. In detail, the amounts of sorghum added in protocols C and D were half those added in protocols A and B (the normal SAB-producing technology). When fermented for about 30 to 35 days, the fermented grains of protocols A and C were, respectively, remixed with *Daqu* and second-distilled SAB (so-called “*Huijiu jiaqu*”, HJJQ) and fermented for about 30 to 40 days. The results showed that the acidities of the final fermented grains of protocols A (2.43 ± 0.09 mmol/10 g) and C (3.18 ± 0.08 mmol/10 g) were lower than those of protocols B (3.71 ± 0.07 mmol/10 g) and D (4.66 ± 0.10 mmol/10 g), while the alcohol contents in the final fermented grains of protocols A (18.33 ± 0.76%) and C (15.33 ± 1.08%) were higher than those of protocols B (5.10 ± 0.85%) and D (1.85 ± 0.62%). No significant differences were observed in the other physicochemical indices among the samples. The HJJQ operation significantly increases the alcohol content and reduces the acidity of the fermented grains but has little influence on the other physicochemical indices during SAB fermentation. Excluding the influence of the HJJQ operation and a half input of sorghum on the EC precursor and EC contents for the fermented grains of protocol B, the linear relationships between the EC content and alcohol (R^2^: 0.4465), citrulline (R^2^: 0.6962), urea (R^2^: 0.4705), and HCN (R^2^: 0.6324) contents were good (all the confidence levels were at 0.05), indicating that these compounds were the dominant EC precursors during SAB fermentation. HJJQ also facilitated the reaction between alcohol and other EC precursors, decreasing EC precursor content and increasing the EC content. KEGG pathway analysis demonstrated that EC precursors were mainly synthesized by alcohol and arginine metabolism. HCN (R^2^: 0.3875 to 0.8198) and alcohol (R^2^: 0.4642 to 0.8423) were the dominant EC precursors during SAB distillation. Overall, the HJJQ operation, especially in protocol C, could significantly reduce the content of EC in base SAB, and the base SAB obtained was of good quality. This, therefore, may be an alternative and effective way to reduce the EC content in base *Baijiu*.

## 1. Introduction

Ethyl carbamate (EC, CAS No. 51-79-6), a colorless, odorless compound naturally present in various fermented foods and beverages such as soybean paste, yogurt, cheese, vinegar, and alcoholic beverages, is regarded as an animal carcinogen that increases the risk of tumor incidence [1]. With increasing research on the carcinogenic mechanisms of EC, it was upgraded from class 2B carcinogen to class 2A carcinogen by the World Health Organization’s International Agency for Research on Cancer (IARC) [2]. Briefly, most of the EC adsorbed in a rodent’s body is hydrolyzed into carbon dioxide, ammonia, and alcohol or excreted in the urine. Approximately 5% of EC is converted by cytochrome P450 into N-hydroxyl amino ethyl formate, α-hydroxy amino ethyl formate, and vinyl ethyl carbamate; the carcinogenicity of the latter is stronger than that of EC and is further converted into ethylene carbamate epoxide by cytochrome P450. N-hydroxyl amino ethyl formate and ethylene carbamate epoxide can damage the double-stranded structure of DNA by binding to DNA and proteins, further causing the occurrence of tumors [3,4].

EC has been widely detected in various alcoholic beverages, including stone-fruit spirits, distilled spirits, rice wine, and beer [5]. Based on the research data, the EC contents in alcoholic beverages were found to be higher than those in other fermented foods [6]. In reality, alcoholic beverages have gradually become the main intake sources of EC due to their widespread consumption all over the world. Concerning the carcinogenicity of EC and public health, many countries have introduced limit levels for EC in alcoholic beverages, these range from 30 μg/L to 1000 μg/L [5,7]. In China, *Baijiu*, produced through a traditional fermentation process, is one of six world-famous distilled spirits, has a several-thousand-year history, and is one of the most-consumed distilled spirits in the world, with an annual consumption of about 6.29 billion liters in the year 2023. *Baijiu* is classified into *Fen*-flavor *Baijiu* (FFB), Strong-aroma *Baijiu* (SAB), *Maotai*-flavor *Baijiu* (MFB), *Feng*-flavor *Baijiu*, Medicinal-flavor *Baijiu*, Miscellaneous-flavor *Baijiu*, Sesame-flavor *Baijiu*, *Chi*-flavor *Baijiu*, Rice-flavor *Baijiu*, and *Te*-flavor *Baijiu* based on its flavor and taste characteristics [8]. In recent decades, extensive research on the EC levels in *Baijiu* has been conducted, and significant differences have been observed in the EC contents in different types of *Baijiu* [3,4]. As reported, the EC contents in base *Baijiu* (freshly distilled *Baijiu*) are ranked as follows: *Feng*-flavor (822.23 μg/L) > *Te*-flavor (339.34 μg/L) > SAB (298.21 μg/L) > Sesame-flavor (269.76 μg/L) > MFB (246.17 μg/L) > FFB (158.24 μg/L) > *Medicinal*-flavor (80.32 μg/L). For bottled *Baijiu* (commercial *Baijiu*), it is ranked as follows: Sesame-flavor (214.13 μg/L) > SAB (191.89 μg/L) > *Feng-flavor* (168.24 μg/L) > *Te*-flavor (93.43 μg/L) > MFB (72.81 μg/L) > *Medicinal*-flavor (65.09 μg/L) > FFB (46.23 μg/L) [9]. It has been shown that the higher the EC content in base *Baijiu*, the higher the EC content in bottled *Baijiu*. The increased consumption and EC content of *Baijiu* may expose consumers to a high EC intake per day and the associated health risks. The margin of exposure (MOE) is often used to assess the EC health risk. Chinese researchers have investigated the effects of daily exposure and the MOE of EC for regular consumers based on the EC contents of three typical *Baijiu* products that occupy over 70% of the *Baijiu* market [7,9]. The daily exposure of EC was confirmed as 40 ng/kg bw/day for *Baijiu* consumers, and SAB consumers displayed a higher intake of about 73.2 ng/kg bw/day, followed by MFB (37.33 ng/kg bw/day) and FFB consumers (7.72 ng/kg bw/day). The calculated mean MOE value for EC is 7500, that of FFB is the highest, at about 38,860, that of MFB is 8036, and that of SAB is the lowest, at about 4098. Based on animal trials, when the MOE value is below 10,000, as the value decreases, the risk of cancer increases. Thus, the EC exposure caused by MFB and SAB consumption poses a significant risk to consumer health. Due to the lack of detailed research and related clinical trials, the Chinese government has not yet established a standard limit for EC in *Baijiu* [10]. Concerning the *Baijiu* consumers’ health, besides limiting the daily consumption of *Baijiu*, it is necessary to reduce the EC content in *Baijiu*.

*Baijiu* production is a multilateral fermentation process involving synchronous saccharification and fermentation with multiple grains, microbes, and enzymes and is influenced by various factors such as fermentation patterns, batching, moisture, acidity, alcohol content, and temperature. Therefore, it is difficult to obtain a more comprehensive formation mechanism for EC during *Baijiu* fermentation. So far, some researchers have found that changes in the urea content are basically simultaneous with changes in the EC content during *Baijiu* fermentation [11]. Correlation analysis between urea, cyanide (HCN), citrulline, and EC showed that urea and HCN are the major EC precursors during *Baijiu* fermentation [12]. Meanwhile, other researchers have concluded that urea and citrulline are the primary EC precursors during *Baijiu* fermentation and that HCN does not participate in EC formation [13]. The EC precursor in *Baijiu* during distillation and storage is commonly thought to be HCN [14,15], but there are still differing opinions and some think that urea and citrulline are the main EC precursors in *Baijiu* during storage [11,16]. This means that more in-depth basic research should be carried out to reveal a more comprehensive formation mechanism for EC during *Baijiu* fermentation and distillation. Recently, based on the preliminary understanding of EC formation during *Baijiu* fermentation and distillation, a number of studies were conducted to reduce the EC content in *Baijiu*; these were mainly focused on the microbial degradation of EC precursors [17,18,19,20], distillation optimization [3,21], and storage condition optimization [1,22,23]. These efforts could significantly control the levels of the main EC precursors and EC content during *Baijiu* fermentation and in base *Baijiu*. However, artificially inoculating strains with special functions into the *Baijiu* production microbial system may negatively affect the microbial community and the metabolic network balance during fermentation and off-flavors may be introduced into the base *Baijiu*.

In the present work, we endeavored to analyze the changes in the physiochemical indices, functional microbes, and EC precursor and EC contents of fermented grains under different fermentation patterns during SAB fermentation and EC precursor and EC contents during distillation to reveal EC’s formation mechanism during these processes and to further provide an effective method for reducing the EC content in base SAB.

## 2. Materials and Methods

### 2.1. Reagents and Materials

Glutinous sorghum and rice husks were purchased from a local market. Fermentation starters (*Daqu*) were self-produced. Urea, arginine, citrulline, o-phthaldialdehyde-3-mercaptopropionic acid, 9-fluorenylmethyl-chloroformate, sodium acetate, triethanol amine, tetrahydroruran, ethanol, acetonitrile, acetic acid, 9-xanthydrol, hydrochloric acid, EC-d5, and EC were of chromatographic grade and purchased from Sigma-Aldrich (St. Louis, MO, USA). Packtest free cyanide WAK-CN kits (the detection range of cyanide by this kit is 0.02 mg/L to 2 mg/L) were obtained from Kyoriso (Osaka, Japan). SLE (diatomite, 3 mL) SPE columns were purchased from Swell Scientific Instruments Co., Ltd. (Chengdu, China). The other reagents were of analytical grade and were obtained from a local reagent supplier.

*Daqu* were self-produced by a traditional fermentation process. Briefly, wheats were ground into powders, the parts passing through sieve with a 840 µm opening size were controlled at below 40%. Thereafter, wheat powders were added with 38% (*w*/*w*) of tap-water, mixed thoroughly, and stood for 5 min to assimilate enough water. Then, they were pressed into bricks, transferred into the fermentation room, and naturally fermented for about 15 days. When fermentation finished, the bricks were transferred into a storeroom and matured for about 3 months before being used for SAB fermentation.

### 2.2. Experiments

The greater the age of the cellar used, the more stable the *Baijiu* fermentation will be and the more representative the experimental results will be. A high production temperature can accelerate the reaction between urea, cyanide, citrulline, and alcohol to synthesize EC [5]. Therefore, to obtain representative results, we selected four cellars with ages of about 100 years at *Baijiu*-making enterprises to conduct our SAB fermentation experiments and produce SAB in the summer season to clarify the formation mechanism of EC during SAB fermentation. All experiments were carried out on an industrial scale using the traditional fermentation process with minor modifications [24]. Briefly, rice husks were steamed for 30 min in a steaming bucket. Glutinous sorghum was ground into powder, and the parts passing through a sieve with a 840 µm opening size were controlled at below 50%. Then, the glutinous sorghum powders were added with 80% (*w*/*w*) of 80 °C water, mixed thoroughly, and left to stand 4 h to assimilate enough water. Then, the glutinous sorghum powders, fermented grains from the former fermentation batch (so-called mother *Zaopei*), and rice husks were thoroughly mixed at a ratio of 5:8:1 (*w*/*w*/*w*) for protocols A and B and at a ratio of 2.5:8:1 (*w*/*w*/*w*) for protocols C and D. Subsequently, the mixed materials were, respectively, steamed for 55 min in a steaming bucket. After being cooled to the required temperature, the steamed materials were thoroughly mixed with 18% *Daqu* (*w*/*w*), transferred into the cellar, and sealed with pit mud to create an anaerobic environment for SAB fermentation. In order to try to facilitate the formation of EC by EC precursors and alcohol, when fermented for about 30 to 35 days, the fermented grains of protocols A (protocol B as blank) and C (protocol D as blank) were, respectively, taken out from the cellar and remixed with 100 kg of *Daqu* and 600 kg of second-distilled SAB, an operation referred to as “*Huijiu jiaqu*” (abbreviated as HJJQ). Afterwards, they were re-transferred into the original cellar and fermented for about 30 to 40 days. When the fermentation finished, the fermented grains from the four protocols were put into a steaming bucket for distilling and collecting base SAB, respectively. In practice, protocol B was the normal SAB-producing technology and protocol A was the normal SAB-producing technology with HJJQ modification. Protocol D was a modified protocol B with half the input of glutinous sorghum. Protocol C was a modified protocol A with half the input of glutinous sorghum.

### 2.3. Sampling

To clarify the EC formation mechanism during SAB fermentation, fermented grains were sampled from the upper layer to lower layer via the five-point sampling method at different fermentation periods. For protocols A and B, the fermented grains were sampled at 0 d, 5 d, 10 d, 15 d, 20 d, 30 d, HJJQ, 40 d, 50 d, 60 d, 70 d, and 80 d and labeled as A and B, respectively. For protocols C and D, the fermented grains were sampled at 0 d, 5 d, 10 d, 15 d, 25 d, 35 d, HJJQ, 45 d, 55 d, and 65 d and labeled as C and D, respectively. The samples were stored at −80 °C until further experiments to determine the physiochemical indices, EC precursors contents, EC contents, and microbial communities.

To investigate the effect of the four protocols on EC precursors and EC contents in base SAB, the base SAB originating from the upper layer and lower layer of the fermented grains of protocols A, B, C, and D were, respectively, collected and labeled as A_1_, B_1_, C_1_, and D_1_ (upper layer) or A_2_, B_2_, C_2_, and D_2_ (lower layer).

During SAB distillation, 100 mL of the base SAB was gathered per minute until the distillation finished to investigate the EC formation mechanism, and a total of 5 SAB distillation processes were conducted. The base SAB samples obtained from the 5 SAB distillation processes were labeled as A SAB, B SAB, C SAB, D SAB, and E SAB, respectively. All base SAB samples were stored at 4 °C until further experiments to detect EC precursors and EC contents.

### 2.4. Determination of Physiochemical Indices

The fermentation temperatures were measured by inserting 5 digital thermometers at different depths into the fermented grains in the cellar. The mean value ± standard deviation (SD) of the 5 temperature measurements collected at the same time was used to represent the fermentation temperature of the grains. The moisture, acidity, reducing sugar contents, and starch and alcohol contents in fermented grains were detected according to a method described previously [25]. The moisture content was determined via the constant weight method. An amount of 20 g of the fermented grains and 200 mL of distilled water were added into 500 mL beakers, and then they were stirred for 30 min. Thereafter, they were filtered with filter papers (Whatman No. 4). The filtrates were used to determine the acidity and reducing sugar content (RS) via alkali titration and Fehling’s reagent titration, respectively. Amounts of 5.00 g of fermented grains and 100 mL of 2% hydrochloric acid (*w*/*w*) were added into 250 mL conical flasks capped with cork, and then they were heated for 30 min at 121 °C to hydrolyze the starch into reducing sugar. The hydrolysates were neutralized with 10% sodium hydroxide (*w*/*w*), diluted to 200 mL with distilled water, and filtered with filter paper. The filtrates were used to determine the total reducing sugar content (TRS). The starch content (ST) was calculated using the equation: ST (%) = (TRS-RS) × 0.9. Amounts of 100 g of fermented grains and 250 mL of distilled water were added into 500 mL round-bottomed flasks and heated to collect 100 mL of distillates. A densimeter was applied to measure the alcohol contents of the distillates using a standard calibration table calibrated at 20 °C as reference.

### 2.5. Determination of EC Precursors and EC Contents

Extracts of fermented grains were prepared based on a previous method [16]. In this process, 10 g fermented grains were added to 20 mL of ultra-pure water and ultrasonically extracted for 1 h in an ice bath. Then, they were centrifuged at 12,000 rpm and 4 °C for 10 min. The supernatants were collected and stored at −20 °C for EC precursor and EC content analysis.

#### 2.5.1. Determination of Arginine and Citrulline Contents

Extracts of fermented grains and base SAB were, respectively, pre-treated with 10% trichloroacetic acid and filtered with a 0.22 μm organic diameter. The arginine and citrulline contents in the extracts of fermented grains and base SAB were determined from the means of high-performance liquid chromatography (HPLC) using an Agilent 1260 series instrument (Agilent, Santa Clara, CA, USA) equipped with an ODS HYPERSIL column (250 × 4.6 mm × 5 μm, ThermoFisher, Waltham, MA, USA) and a variable wavelength UV/Vis detector after pre-column derivatization with o-phthaldialdehyde-3-mercaptopropionic acid and 9-fluorenylmethyl-chloroformate, as described previously [16]. HPLC analyses were performed as follows: the mobile phase consisted of solutions A and B. Solution A (1 L) consisted of 2.2 g of sodium acetate, 220 mL triethanol amine, and 6 mL tetrahydroruran, and the pH was adjusted to 7.2 with 5% acetic acid. Solution B consisted of 1.8 g of sodium acetate, 320 mL of ethanol, and 400 mL of acetonitrile, and the pH was adjusted to 7.0 with 5% acetic acid. The mobile phase was set at a constant flow rate of 1.5 mL/min. The oven temperature was 40 °C, and the detection wavelength was 338 nm.

#### 2.5.2. Determination of Cyanide Contents

The amount of cyanide (HCN) in the extracts of fermented grains and base SAB was detected using packtest free cyanide WAK-CN kits according to the manufacturer’s instructions.

#### 2.5.3. Determination of Urea Contents

Extracts of fermented grains and base SAB were, respectively, derived with 9-xanthydrol and hydrochloric acid in the dark. Then, the derived samples were studied via HPLC equipped with a C18 column (250 × 4.6 mm × 5 μm, Agilent, CA, USA) and a fluorescence detector according to a previously described method [16]. A total of 10 μL of the derived samples were injected for HPLC analysis. HPLC analyses were performed as follows: the mobile phase was 20 mM sodium acetate at a constant flow rate of 1.0 mL/min. The oven temperature was 35 °C, and the excitation and emission wavelengths were 213 nm and 308 nm, respectively.

#### 2.5.4. Determination of Alcohol Contents

The alcohol contents (%, *v*/*v*) in base SAB were detected by volume by taking 100 mL of base SAB in a measuring cylinder. A densimeter was applied to measure the alcohol content using a standard calibration table calibrated at 20 °C as reference [25].

#### 2.5.5. Determination of EC Contents

The EC in extracts of fermented grains and base SAB was extracted with SLE (diatomite, 3 mL) SPE columns by following the manufacturer’s instructions. The EC contents were determined using an GC-MS (QP2020 NX, SHIMADZU, Japan) equipped with an HP-innowax capillary column (60 m × 0.25 µm × 0.25 µm, Agilent, CA, USA), consulting a previous method [26]. A total of 1 μL of the EC extracts was injected for GC-MS analysis. The GC operation conditions were as follows. The injector temperature was 250 °C. High-purity helium (99.999%) was used as a carrier gas at a constant flow rate of 1 mL/min. Splitless mode was used. The oven temperatures were programmed as follows: held at 100 °C for 3 min, then raised to 130 °C at a rate of 4 °C/min. Then, it was raised to 170 °C at a rate of 8 °C/min, followed by an increase to 220 °C at a rate of 10 °C/min and held for 10 min. The mass spectrometer was operated in selected ion monitoring mode (SIM), major ion *m/z* 62, 74, and 89 were monitored and *m/z* 62 was chosen as the quantitative ion. The ion source, quadrupole mass filter, and transfer line temperatures were maintained at 230 °C, 150 °C, and 250 °C.

### 2.6. Microbial Community Analysis

#### 2.6.1. DNA Extraction, Library Construction, and Metagenomic Sequencing

Total genomic DNA was extracted from the fermented grains according to a previous method [27]. To ensure that adequate amounts of high-quality genomic DNA had been extracted, the content and purity of genomic DNA were determined with a TBS-380 and NanoDrop2000, respectively. The quality of the extracted genomic DNA was examined via 1% agarose gel electrophoresis.

Then, 2 μg of genomic DNA was fragmented into an average size of 350 bp using Covaris M220 (Majorbio Bio-Pharm Technology Co., Ltd., Shanghai, China) for constructing a paired-end library. Genomic DNA paired-end libraries were constructed via NEXTFLEX^®^ Rapid DNA-Seq (Bioo Scientific, Austin, TX, USA). Adapters containing the full complement of sequencing primer hybridization sites were ligated to the blunt end of genomic DNA fragments. Paired-end sequencing was conducted on an Illumina NovaSeq6000 (Illumina Inc., San Diego, CA, USA) at Majorbio BIo-Pharm Technology Co., Ltd. (Shanghai, China) using NovaSeq6000 S4 Reagent Kit v1.5 (300 cycles) following the manufacturer’s instructions (www.illumina.com, 30 June 2020).

#### 2.6.2. Sequence Quality Control and Genome Assembly

The sequence data were analyzed on the free online Majorbio Cloud Platform (www.majorbio.com, 2 June 2024). Concisely, the paired-end Illumina reads were trimmed of adaptors and low-quality reads with a length < 150 bp or a quality value < 20 were removed using fastp [28] (https://github.com/OpenGene/fastp, version 0.23.0, 2 June 2024). Metagenomics data were assembled using MEGAHIT [29] (https://github.com/voutcn/megahit, version 1.1.2, 2 June 2024), which makes use of succinct de Bruijin graphs. Contigs with a length > 300 bp were selected as the final assembling results. Then, the assembled contigs were applied for further gene prediction and annotation.

#### 2.6.3. Gene Prediction, Taxonomy, and Functional Annotation

Open reading frames (ORFs) from each assembled contig were predicted via Prodigal [30]/MetaGene [31] (http://metagene.cb.k.u-tokyo.ac.jp/, 2 June 2024). The predicted ORFs with a length ≥ 100 bp were retrieved and translated into amino acid sequences using the NCBI translation table (http://www.ncbi.nlm.nih.gov/Taxonomy/taxonomyhome.html/index.cgi?chapter=tgencodes#SG1, 2 June 2024).

Non-redundant gene catalogs were constructed using CD-HIT [32] (http://www.bioinformatics.org/cd-hit/, version 4.6.1, 2 June 2024), with 90% sequence identity and 90% coverage. High-quality reads with a length ≥ 300 bp were aligned to the non-redundant gene catalogs for calculating gene abundance, with 95% identity by SOAPaligner [33] (http://soap.genomics.org.cn/, version 2.21, 2 June 2024).

Representative sequences of non-redundant gene catalogs were aligned to the NR database with an e-value ≤ 1 × 10^−5^ using Diamond [34] (http://www.diamondsearch.org/index.php, version 0.8.35, 2 June 2024) for taxonomy annotations. KEGG annotations were carried out via Diamond against the Kyoto Encyclopedia of Genes and Genomes database (http://www.kegg.jp, 2 June 2024), with an e-value ≤ 1 × 10^−5^.

### 2.7. Sensory Properties Analysis

The sensory properties of A_1_, A_2_, B_1_, B_2_, C_1_, C_2_, D_1_, and D_2_ were analyzed by a well-trained panel with six national judges (3 females and 3 males between 25 and 30 years of age) according to the methods described in a professional book [35]. A total of nine evaluation dimensions, namely, transparency, aroma, softness, sweetness, fullness, pure taste, aftertaste, stale taste, and features, were applied to determine the quality of base SAB. The one-hundred-points method was used and the score range of each evaluation dimension is shown in Table S1. If the total sensory score of SAB was one hundred, this indicated that the quality of this sample was excellent.

### 2.8. Statistical Analysis

All tests were performed in triplicate and the data were indicated as the mean value ± SD. Figures were generated with OriginPro 9.1 software (Origin, Northampton, MA, USA) and Illustrator 2024 (Adobe, San Jose, CA, USA). Significant differences in the HCN and EC contents in base SAB were evaluated by means of one-way analysis of variance (ANOVA) with Duncan’s multiple comparison test (*p* < 0.05) using SPSS 19.0 software (SPSS Inc., Chicago, IL, USA). The data for sensory properties were indicated as the average of the scores from six judges.

## 3. Results and Discussion

### 3.1. Analysis of Physicochemical Indices

**Figure 1:**
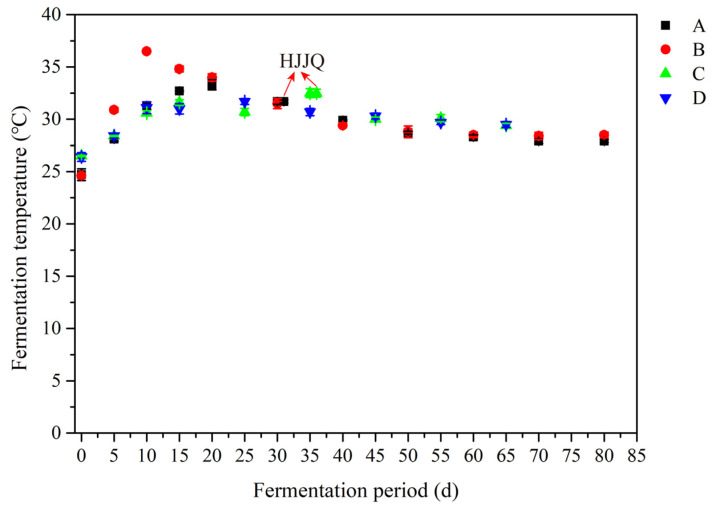
Changes in fermentation temperatures of fermented grains during SAB fermentation. A: fermented grains produced by protocol A during fermentation, B: fermented grains produced by protocol B during fermentation, C: fermented grains produced by protocol C during fermentation, D: fermented grains produced by protocol D during fermentation. HJJQ: fermented grains sampled after the HJJQ operation was finished.

To determine the effect of protocols A, B, C, and D on physicochemical indices of fermented grains during SAB fermentation, the fermentation temperature, moisture, acidity, reducing sugar contents, and starch and alcohol contents of fermented grains were analyzed (Figure 1). The physicochemical indices can reflect the SAB fermentation state and degree, as well as the microbial activity during the fermentation [36,37]. As fermentation proceeded, the fermentation temperatures increased from about 25.50 °C to a peak value of nearly 35 °C at 15 d due to microbial growth, reproduction, and metabolism. When fermentation proceeded to 30 and 35 days for protocols A and C, respectively, the HJJQ operation was conducted and the resulting increase in the dissolved oxygen content in the fermented grains facilitated increased microbial growth, reproduction, and metabolism, further leading to fermentation temperatures slightly increasing to nearly 32 °C and remaining at 28 °C to 29.5 °C until the end of the process. Overall, the fermentation temperatures remained at a high value and may accelerate EC formation during SAB fermentation. For the first 30 to 35 days, also referred to as the primary fermentation period, the fermented grain temperatures of protocols A and B were higher than those of protocols C and D. Meanwhile, for the last 30 to 45 days, also referred to as the volatile formation period, the opposite trends were observed. During the primary fermentation period, the higher the amount of glutinous sorghum added, the higher the rate of increase in the fermentation temperature, while during the volatile formation period, the higher the rate of decrease in the fermentation temperature. It was observed that the starch content of the initial fermented grains directly or indirectly influenced the whole SAB fermentation process.

**Figure 2:**
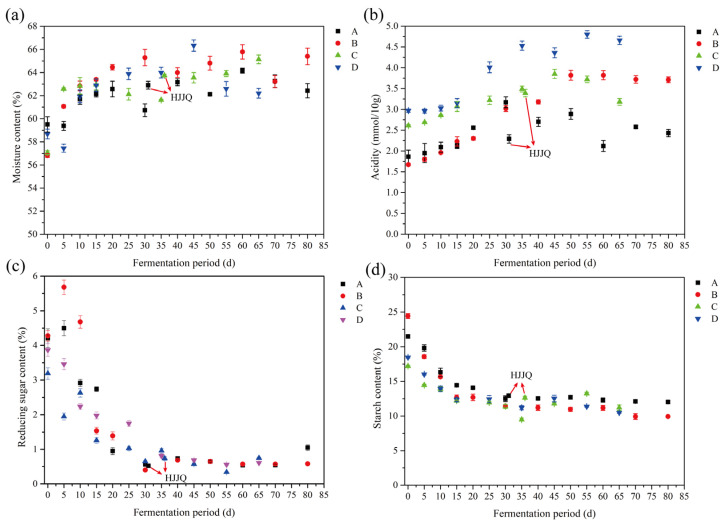
Changes in physicochemical indices of fermented grains during SAB fermentation: (**a**) moisture content, (**b**) acidity, (**c**) reducing sugar content, (**d**) starch content. A: fermented grains produced by protocol A during fermentation, B: fermented grains produced by protocol B during fermentation, C: fermented grains produced by protocol C during fermentation, D: fermented grains produced by protocol D during fermentation. HJJQ: fermented grains sampled after the HJJQ operation was finished.

The changes in the moisture, acidity, and reducing sugar and starch contents of fermented grains during SAB fermentation are depicted in Figure 2. During SAB fermentation, nutrients such as starch, proteins, and cellulose in the fermented grains were metabolized into energy, water, CO_2_, alcohol, acid, and other volatile compounds, and thus the moisture content of fermented grains exhibited an increasing trend for the first 30 to 35 days. As fermentation proceeded, the content of oxygen dissolved in the fermented grains decreased, while the acidity and alcohol contents of the fermented grains increased and microbial activities were inhibited. Therefore, the moisture contents presented a fluctuating state for the last 30 to 45 days (Figure 2a). The acidity of the fermented grains increased as the fermentation processed and that of protocols C and D was higher than protocols A and B (Figure 2b). Compared with protocols B and D, the HJJQ operation used in protocols A and C significantly reduced the acidity of the fermented grains, which may have resulted from the esterification action between the alcohol in second-distilled SAB added and the acids existing in fermented grains. The starch contents of the initial fermented grains of protocols A (21.17 ± 0.22%) and B (24.41 ± 0.36%) were higher than that of protocols C (17.20 ± 0.12%) and D (18.46 ± 0.11%) (Figure 2c). The starch was converted into dextrin, maltose, and glucose during SAB fermentation and, on this count, the reducing sugar contents of the initial fermented grains of protocols A (4.21 ± 0.27%) and B (4.28 ± 0.15%) were higher than those of protocols C (3.19 ± 0.17%) and D (3.70 ± 0.18%) (Figure 2d). Similar trends were observed in the starch and reducing sugar contents during SAB fermentation; their contents consistently decreased until about 30 days and then became flat. Overall, compared with protocols B and D, the HJJQ operation in protocols A and C had little effect on the changes in moisture and the reducing sugar and starch contents during SAB fermentation.

### 3.2. Changes in EC Precursors and EC Contents of Fermented Grains During SAB Fermentation

**Figure 3:**
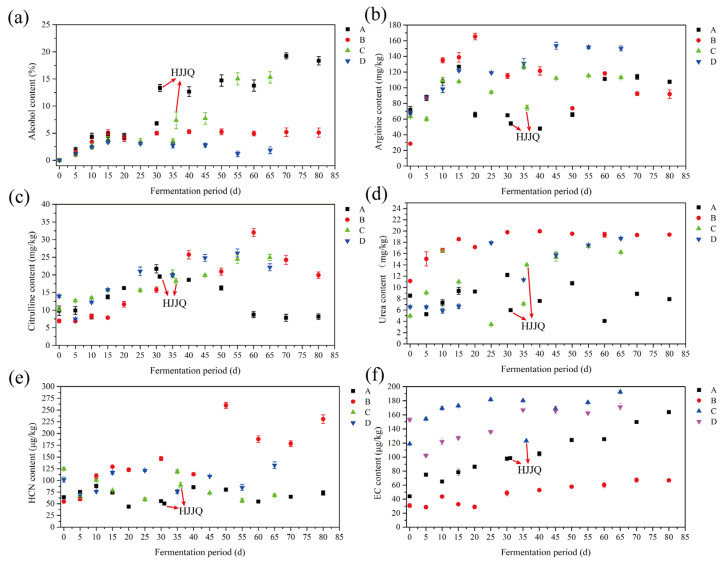
Changes in the EC precursors and EC contents of fermented grains during SAB fermentation: (**a**) alcohol content, (**b**) arginine content, (**c**) citrulline content, (**d**) urea content, (**e**) HCN content, (**f**) EC content. A: fermented grains produced by protocol A during fermentation, B: fermented grains produced by protocol B during fermentation, C: fermented grains produced by protocol C during fermentation, D: fermented grains produced by protocol D during fermentation. HJJQ: fermented grains sampled after the HJJQ operation was finished.

As previously reported, alcohol, citrulline, urea, and HCN are considered as the main EC precursors in fermented foods and alcoholic beverages [4,5]. The potential EC precursors (alcohol, arginine, citrulline, urea, and HCN) and EC contents of the fermented grains sampled after different fermentation periods were analyzed to reveal the EC formation mechanism during SAB fermentation (Figure 3).

The alcohol contents continuously increased to a peak value at 15 d; in this period, the alcohol contents of protocols A and B were higher than those of protocols C and D, which may have resulted from the different reducing sugar contents of the fermented grains (Figure 2d). When fermented to 30 to 35 days, compared with protocols B and D, when the HJJQ operation of protocols A and C was conducted, the alcohol in the second-distilled SAB added significantly increased the alcohol contents in the fermented grains of protocols A and C, which may further promote EC precursors forming EC and lead to an increase in the EC content [12].

The arginine and citrulline contents of the fermented grains were determined and are shown in Figure 3b,c. The results demonstrated that large amounts of arginine and citrulline were detected in the initial fermented grains, indicating that these compounds remained in the residues after distillation, and mother *Zaopei* is also the main source of arginine and citrulline. As fermentation proceeded, the arginine and citrulline contents of the fermented grains of protocols B and D exhibited similar trends, consistently increasing until about 30 days and thereafter tending toward stability. For the fermented grains of protocols A and C, the arginine contents increased until the HJJQ operation was conducted, then slightly declined and rose, becoming stable. The citrulline contents continuously increased until the HJJQ operation was conducted, and then the contents declined toward becoming stable. The arginine in the fermented grains originated from proteins decomposed by microbes and proteinases. During the primary fermentation period, the microbial growth, reproduction, and decomposition metabolism were vigorous and proteins could be rapidly degraded into amino acids, thus leading to an increase in arginine content. As fermentation proceeded, lactic acid bacteria gradually became the dominant bacteria, and the arginine that accumulated may have been metabolized into citrulline through the arginine deiminase pathway (ADI) of lactic acid bacteria, inducing an increase in citrulline content [4]. When fermentation finished, the arginine and citrulline contents in the fermented grains of protocol C (113.15 ± 1.18 mg/kg and 24.95 ± 0.90 mg/kg, respectively) were higher than those of protocol A (107.46 ± 1.22 mg/kg and 8.14 ± 0.8 mg/kg, respectively). A similar phenomenon was also observed in the fermented grains of protocols D (150.22 ± 3.20 mg/kg and 22.16 ± 1.00 mg/kg, respectively) and B (91.85 ± 5.50 mg/kg and 19.91 ± 0.90 mg/kg, respectively). Compared with protocols B and D, after the HJJQ operation was performed in protocols A and C, the alcohol contents remarkably increased, which would facilitate the reaction between alcohol and citrulline, significantly decreasing the citrulline content and increasing the EC content in return.

As is known, urea mainly originates from sorghum and arginine metabolism by active yeasts during fermentation and greatly contributes to EC formation [14]. The urea contents of the fermented grains were determined and are depicted in Figure 3d. According to the physicochemical properties (boiling point > 190 °C), urea cannot be evaporated into distillate during distillation [38] and remains in the residues. As with arginine and citrulline, mother *Zaopei* was also a source of urea. The urea contents in the fermented grains of protocols A, C, and D presented similar trends, increasing until about 30 days, then decreasing and thereafter increasing to a flat level. As for the fermented grains of protocol B, the urea contents continuously increased for about 30 days and then remained at a stable level. After fermentation was finished, the urea contents in the fermented grains of protocols B (19.37 ± 0.04 mg/kg) and D (18.68 ± 0.12 mg/kg) were the highest, followed by protocol C (16.25 ± 0.08 mg/kg), whereas that of protocol A (7.93 ± 0.22 mg/kg) was the lowest. Compared with protocol B, HJJQ in protocol A led to a decrease in urea content; however, as fermentation proceeded, the urea content gradually increased to a fluctuating state.

As previously reported, HCN is mainly produced by decomposing cyanogenic glycosides from various materials via enzymatic action or the acid thermal decomposition and metabolism of arginine during *Lactobacilli* fermentation [4,39]. The HCN in fermented grains could be easily evaporated into base SAB due to its lower boiling point (about 26 °C). However, after distillation, some HCN remained in the initial fermented grains of protocols A, B, C, and D (54.67 ± 2.17 μg/kg to 125.03 ± 3.13 μg/kg, Figure 3e), which may have been produced by the thermal decomposition of urea [12], also demonstrating that mother *Zaopei* was also a source of HCN. The HCN contents of the initial fermented grains of protocols A (63.97 ± 2.54 μg/kg) and B (54.67 ± 2.17 μg/kg) were remarkably lower than those of protocols C (125.03 ± 3.13 μg/kg) and D (101.87 ± 5.35 μg/kg). During SAB fermentation, the HCN contents in the fermented grains of protocols A and C increased at first and then decreased, and after HJJQ was performed, the HCN contents continuously decreased until the end of the process. The alcohol in the second-distilled SAB added during the HJJQ operation in protocols A and C may facilitate the reaction rate between HCN and ethanol during SAB fermentation, finally reducing the HCN contents in the fermented grains of protocols A and C [20,38]. The HCN content in the fermented grains of protocol B increased at first and then remained at a high level in the whole fermentation process. For protocol D, the HCN content first increased and then remained in a fluctuating state. Compared with protocols B (230.67 ± 9.1 μg/kg) and D (132.59 ± 7.16 μg/kg), HCN contents in the final fermented grains of protocols A (73 ± 4.68 μg/kg) and C (68.09 ± 3.17 μg/kg) were relatively lower.

In summary, the contents EC precursors grew with increasing SAB fermentation time, and mother *Zaopei* was also one of the main sources of EC precursors. To investigate the formation mechanism of EC, changes in EC contents of the fermented grains were analyzed via GC-MS (Figure 3f). The EC contents of the initial fermented grains were relatively high due to only a low percentage of EC evaporating into the base SAB owing to its high boiling point (about 184 °C) [12]. The EC contents of the fermented grains constantly increased until fermentation was completed. Compared with protocol B, the EC content in the fermented grains of protocol A were relatively higher. The EC content in the fermented grains of protocol C were also higher than those of protocol D. The EC contents in the initial and final fermented grains of protocols C (118.87 ± 0.69 μg/kg and 192.42 ± 2.03 μg/kg, respectively) and D (153.05 ± 2.98 μg/kg and 171.44 ± 4.5 μg/kg, respectively) were higher than those of protocols A (44.31 ± 0.67 μg/kg and 163.83 ± 1.18 μg/kg, respectively) and B (30.89 ± 2.89 μg/kg and 66.82 ± 2.52 μg/kg, respectively). Compared with the EC contents in the initial fermented grains, the increments in the EC contents of the final fermented grains of protocols A and C were higher than those of protocols B and D. It was suggested that mother *Zaopei* was also the main source of EC and that the HJJQ operation could significantly facilitate the reaction between EC precursors and improve the EC contents in fermented grains.

### 3.3. Correlation Analysis Between EC, Fermentation Temperature, and EC Precursors During SAB Fermentation

**Figure 4:**
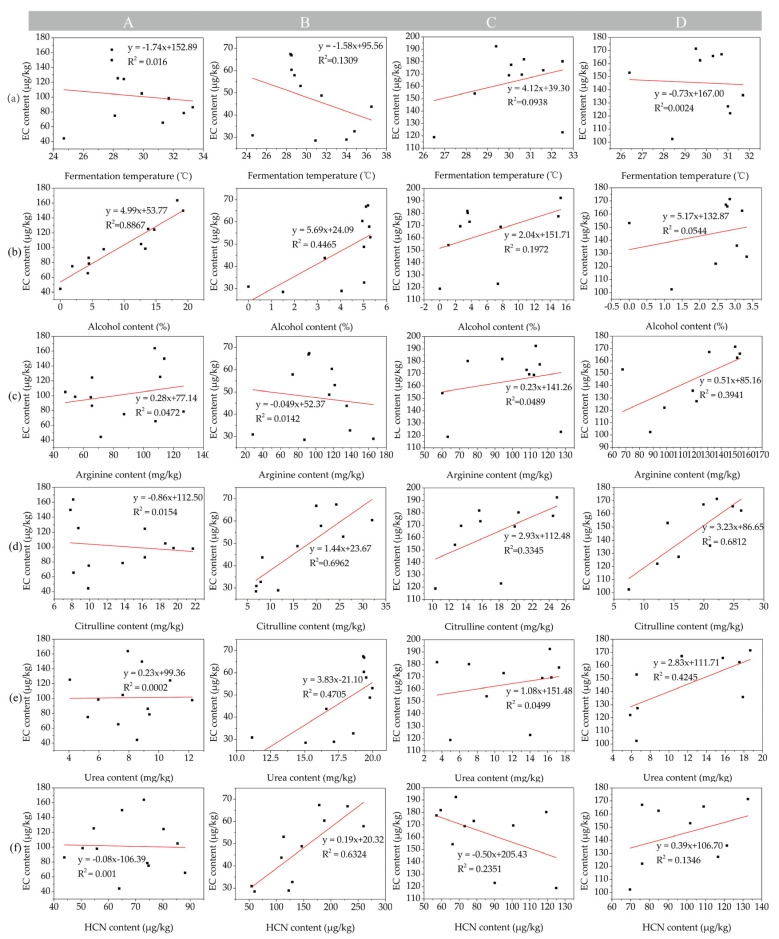
Correlations between EC and its precursors in the fermented grains during SAB fermentation. (**a**) EC and fermentation temperature, (**b**) EC and alcohol content, (**c**) EC and arginine content, (**d**) EC and citrulline content, (**e**) EC and urea content, (**f**) EC and HCN content. A: fermented grains produced by protocol A during fermentation, B: fermented grains produced by protocol B during fermentation, C: fermented grains produced by protocol C during fermentation, D: fermented grains produced by protocol D during fermentation. The squares represent the fermented grains sampled during fermentation. And the red lines represent the linear relationships between EC and EC precursors.

The major contributors to EC formation in fermented foods and alcoholic beverages have been known for decades, but due to the complexity of *Baijiu* solid-state fermentation, there is still controversy regarding the precursors and formation mechanism of EC during *Baijiu* fermentation. Linear regression and bi-variate correlation analysis was conducted based on the mean values of fermentation temperature, EC precursor content, and EC content to analyze the relationships between EC content, fermentation temperature, and EC precursor content during SAB fermentation. As shown in Figure 4, the results show that no significant correlations were observed between the EC content and fermentation temperature (R^2^: 0.016 to 0.1309), as well as the arginine content (R^2^: 0.0142 to 0.0489). For the fermented grains of protocols A, B, and C, the EC contents were positively correlated with the amount of alcohol (R^2^: 0.8867, 0.4465 and 0.1972, respectively); the confidence level was at 0.05. The alcohol content in the fermented grains of protocol D was relatively low, while the EC content in the fermented grains of protocol D was relatively high, which may have been mainly contributed by mother *Zaopei* and may have led to the correlation between the EC content and alcohol content becoming weakened (R^2^: 0.0544). For the fermented grains of protocols B, C, and D, the EC contents were positively correlated with citrulline (R^2^: 0.6962, 0.3345, and 0.6812, respectively); the confidence level was at 0.05. For the fermented grains of protocols B and D, the EC contents were also positively correlated with urea (R^2^: 0.4705 and 0.4245, respectively); the confidence level was at 0.05. For the fermented grains of protocol B, the EC content was also positively correlated with HCN (R^2^: 0.6324); the confidence level was also at 0.05. When HJJQ was performed, second-distilled SAB was added into the fermented grains of protocols A and C and alcohol contents in the fermented grains of protocols A and C significantly increased. The reaction rates between alcohol and citrulline, alcohol and urea, and alcohol and HCN may be accelerated to form EC, resulting in the citrulline, urea, and HCN contents decreasing and the EC contents increasing. This may lead to the correlations between EC and urea, citrulline, or HCN being weakened. Excluding the influence of the HJJQ operation and the half input of sorghum on the EC precursor and EC contents and correlation analysis, for the fermented grains of normal SAB-producing technology (protocol B), the linear relationships between the EC content and alcohol (R^2^: 0.4465), citrulline (R^2^: 0.6962), urea (R^2^: 0.4705), and HCN (R^2^: 0.6324) were good; all the confidence levels were at 0.05. It was indicated that the HJJQ operation and half input of sorghum could influence the correlations between the EC precursors and EC and that the dominant precursors of EC during SAB fermentation include alcohol, citrulline, urea, and HCN.

### 3.4. Changes in Microbial Communities During SAB Fermentation

Alcohol, urea, citrulline, and HCN all contribute to EC formation during SAB fermentation. Genomic DNA extracted from fermented grains was subjected to metagenomic sequencing to explore the influence of microbial communities on EC precursor formation. A total of 459 Gbp of raw data were obtained, cleaned, pooled, and assembled de novo. Over ninety-five percent of the total raw reads passed quality control, indicating that the sequencing quality had reliable data throughput and can be used for subsequent analysis.

#### 3.4.1. Changes in Microbial Diversity During SAB Fermentation

**Figure 5:**
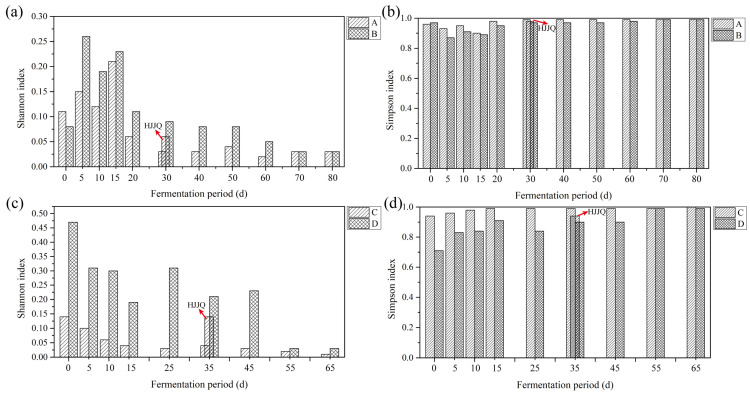
Diversity indices of the microbes present in the fermented grains during SAB fermentation. (**a**,**c**) Shannon index, (**b**,**d**) Simpson index. A: fermented grains produced by protocol A during fermentation, B: fermented grains produced by protocol B during fermentation, C: fermented grains produced by protocol C during fermentation, D: fermented grains produced by protocol D during fermentation. HJJQ: fermented grains sampled after the HJJQ operation was finished.

The Shannon and Simpson indices are usually applied to estimate the microbial diversity of environmental samples. To investigate the effects of protocols A, B, C, and D on microbial diversity during SAB fermentation, the Shannon and Simpson indices were calculated (Figure 5). As fermentation proceeded, the Shannon indices of microbes in the fermented grains of protocols A and B first increased and then decreased to become stable, while those of protocols C and D continuously decreased to become stable. Notably, after the HJJQ operation of protocols A and C was finished, the Shannon indices reached a stable state early. The Simpson indices constantly increased to a stable state in the fermentation process. In particular, the Simpson indices of microbes in the fermented grains of protocols A and C reached a stable level after HJJQ was performed, while those of protocols B and D increased to a stable state after fermented for 55 to 60 days. The results demonstrated that the microbial diversity showed a decreasing trend and that the microbial community structures gradually tended to stabilize as fermentation proceeded. The HJJQ operation was beneficial for promoting the stabilization of the microbial structure.

#### 3.4.2. Changes in Microbial Community During SAB Fermentation

**Figure 6:**
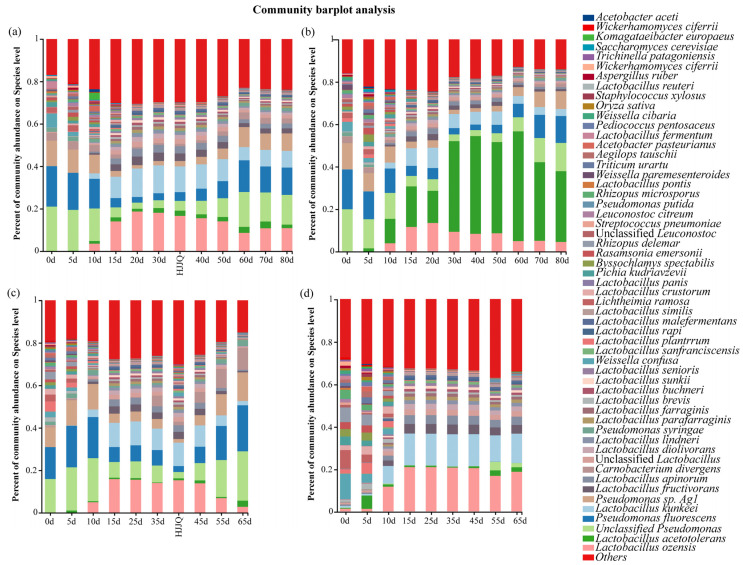
Microbial communities present in the fermented grains during SAB fermentation at the species level: (**a**) fermented grains produced by protocol A during fermentation, (**b**) fermented grains produced by protocol B during fermentation, (**c**) fermented grains produced by protocol C during fermentation, and (**d**) fermented grains produced by protocol D during fermentation. HJJQ: fermented grains sampled after the HJJQ operation was finished.

The microbial communities of the fermented grains during the fermentation process were analyzed at the species level (relative abundance (RA) > 1%); the results are shown in Figure 6 and Table S2. The results suggested that microbial species with a high RA mainly fell into the *Lactobacillus* (RA: 3.03% to 73.08%), *Carnobacterium* (RA: 0% to 10.23%), *Weissella* (RA: 0.04% to 14.42%), *Leuconostoc* (RA: 0.03% to 3.28%), *Pseudomonas* (RA: 0.01% to 54.72%), *Rasamsonia* (RA: 0.01% to 3.38%), *Pichia* (RA: 0.02% to 4.03%), *Byssochlamys* (RA: 0.01% to 3.72%), *Lichtheimia* (RA: 0.01% to 10.75%), and *Rhizopus* (RA: 0.02% to 11.16%) genera under the phyla of *Bacillota*, *Pseudomonadota*, *Ascomycota*, and *Mucoromycota*, respectively. The RA of lactic acid bacteria was the highest, followed by *Pseudomonas*. As shown in Table S2, among the lactic acid bacteria, the RA of *Lactobacillus acetotolerans*, *Lactobacillus ozensis*, *Lactobacillus kunkeei*, *Lactobacillus fructivorans*, *Lactobacillus apinorum*, and *Lactobacillus diolivorans* was relatively high. The RA of unclassified *Pseudomonas*, *Pseudomonas fluorescens, Pseudomonas* sp. Ag1, and *Pseudomonas syringaes* was relatively high. The other species with a high RA included *Carnobacterium divergens, Weissella confusa*, *Rasamsonia emersonii*, *Lichtheimia ramosa*, *Pichia kudriavzevii*, *Leuconostoc citreum, Rhizopus delemar*, and *Rhizopus microsporus*. These microbes detected in the fermented grains were also detected in the *Daqu* used for *Baijiu* fermentation and during other *Baijiu* fermentations, indicating that they may play important roles in *Daqu* and *Baijiu* fermentation, such as enzyme hydrolysis and volatile compound biosynthesis [40,41,42,43]. As fermentation proceeded, the RA of lactic acid bacteria first increased for about 30 days and then declined until the end of the process, while the opposite trends were observed in the RA of *Pseudomonas* and *Carnobacterium divergens*; the RA of the other species consistently declined until fermentation finished. Apparently, changes in the RA of all microbes were bounded by 20 days, and there may be some differences in the metabolic pathways contributed to by microbes before and after this boundary.

### 3.5. Analysis of the Microbial Synthesis Pathways for EC Precursors in Fermented Grains During SAB Fermentation

Microbial genes aligned with the KEGG database were classified into five levels of pathways based on the metagenomic sequencing data (Figure S1). The results demonstrated that carbohydrate metabolism, amino acid metabolism, global and overview maps, energy metabolism, nucleotide metabolism, biosynthesis of other secondary metabolites, xenobiotics biodegradation and metabolism, lipid metabolism, and signal transduction were the dominant KEGG pathways in the fermented grains during SAB fermentation.

**Figure 7:**
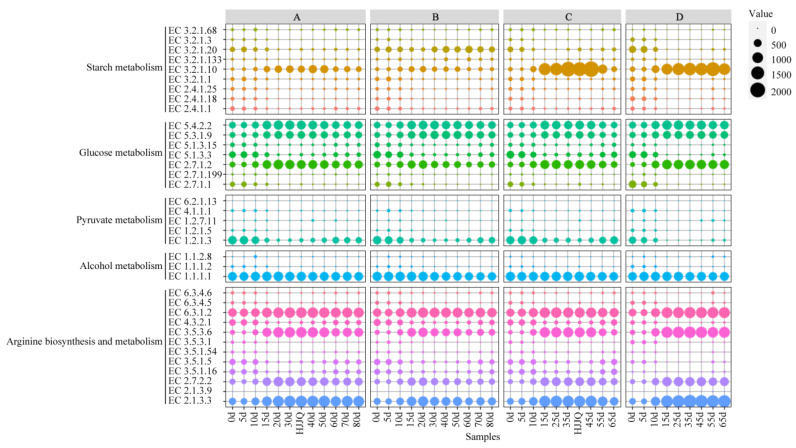
Changes in the coding genes abundances of key enzymes involved in the metabolic pathways of EC precursors during SAB fermentation. A: fermented grains produced by protocol A during fermentation, B: fermented grains produced by protocol B during fermentation, C: fermented grains produced by protocol C during fermentation, D: fermented grains produced by protocol D during fermentation. HJJQ: fermented grains sampled after the HJJQ operation finished. The larger the circle is, the higher coding gene abundance of the key enzyme.

**Figure 8:**
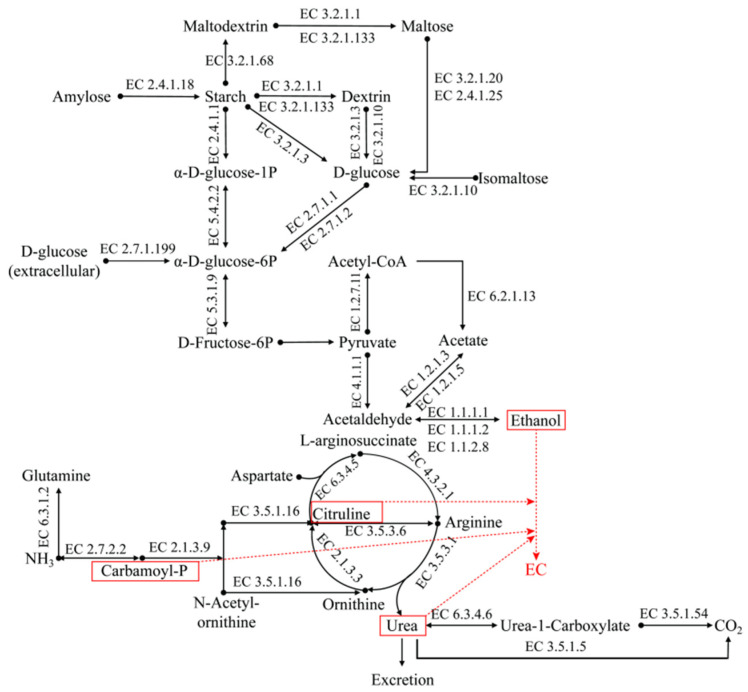
The metabolic pathways of EC precursors during SAB fermentation.

Analysis of EC formation showed that alcohol, urea, and citrulline were the main EC precursors during SAB fermentation. To explore the microbial synthesis pathways of these precursors, KEGG annotations were carried out via Diamond against the Kyoto Encyclopedia of Genes and Genomes database, with an e-value ≤ 1 × 10^−5^. The KO numbers of KEGG annotations were compared with the KEGG mapper database to retrieve metabolic pathways and the codes of representative enzymes. A total of 36 key enzymes were selected for their potential participation in the substrate degradation and EC precursor accumulation, and they were classified into five function assemblies: starch and sucrose metabolism, glycolysis/gluconeogenesis, pyruvate metabolism, alcohol metabolism, and arginine biosynthesis and metabolism (Figure 7). Based on the codes of the 36 key enzymes, the non-redundant gene catalogs were reconstructed and used to obtain microbial taxonomy and functional annotations, the coding genes abundances of key enzymes (Table S3), and the species and functional contributions to key enzymes coding genes (Table S4) on the free online Majorbio Cloud Platform (www.majorbio.com, 2 June 2024). The changes in the coding genes abundances of the key enzymes involved in the metabolic pathways of EC precursors during SAB fermentation are depicted in Figure 7. In general, higher coding gene abundances for key enzymes may result in higher enzyme expression and higher abundances for key enzymes [44]. The microbial synthesis pathways of EC precursors during SAB fermentation are visualized in Figure 8. Changes in the functional contributions of microbes to the coding genes of the 36 key enzymes involved in the microbial synthesis pathways of EC precursors are depicted in Figures S2–S6.

Based on the coding gene abundances of the 36 key enzymes (Figure 7) and FCs of the microbes to the coding gene abundances of the 36 key enzymes (Figures S2–S6), the metabolic pathways of EC precursors (Figure 8) were analyzed in detail. For starch metabolism, as fermentation proceeded, *P.* sp. Ag1 and *L. plantarum* were the potential primary microbes degrading starch or maltodextrin into dextrin and maltose via alpha-amylase (EC 3.2.1.1) and maltogenic alpha-amylase (EC 3.2.1.133). *Acetobacter pasterurians* could also degrade starch and dextrin into glucose using glucoamylase (EC 3.2.1.3), while *L*. *fermentum*, *L*. *plantarum*, *L*.*citreum*, and *Saccharomyces cerevisiae* mainly converted dextrin into glucose via oligo-1,6-glucosidase (EC 3.2.1.10). After starch, maltodextrin, and dextrin were degraded into maltose, it was further metabolized into glucose by *L*. *brevis*, *L*. *plantarum*, and *Aspergillus oryzae* using alpha-glucosidase (EC 3.2.1.20) and 4-alpha-glucanotransferase (EC 2.4.1.25). Overall, starch was mainly degraded into dextrin and maltose, then further degraded into glucose, applying energy for microbial growth, reproduction, and metabolism. During the first 10 days of fermentation, the abundances of alpha-amylase, alpha-glucosidase, 4-alpha-glucanotransferase, oligo-1,6-glucosidase, isoamylase (EC 3.2.1.68), and glycogen phosphorylase (EC 2.4.1.1) were relatively high. They could rapidly decompose starch into dextrin, maltose, glucose, and α-D-glucose-1P, resulting in the starch content decreasing quickly and the glucose content remaining at a high level. In the middle and late stages of fermentation, the abundances of oligo-1,6-glucosidase and alpha-glucosidase were relatively high, and they could decompose dextrin and maltose into glucose. In this period, the generation rate and consumption rate of glucose may tend to be consistent, and the glucose contents of the fermented grains tended to be stable and remained at a relatively low level.

After starch, maltodextrin, dextrin, and maltose were decomposed into D-glucose or α-D-glucose-1P, α-D-glucose and β-D-glucose were converted into each other by *L*. *fermentum*, *L*. *plantarum*, *L*. *sanfranciscensis*, and unclassified *Lactobacillaceae* through aldose 1-epimerase (EC 5.1.3.3) during SAB fermentation. D-glucose was metabolized into D-glucose-6P by hexokinase (EC 2.7.1.1) and glucokinase (EC 2.7.1.2), which were mainly secreted by *L*. *fermentum*, *L*. *plantarum*, and *P*. *fluorescens*. α-D-glucose-1P was converted into D-glucose-6P by *L*. *plantarum*, *P*. *fluorescens*, *P*. sp. Ag1, and unclassified *Leuconostoc* via phosphoglucomutase (EC 5.4.2.2). During SAB fermentation, D-glucose-6P was mainly converted into D-fructose-6P by unclassified *Pseudomonas* via glucose-6-phosphate isomerase (EC 5.3.1.9), and the D-fructose-6P subsequently entered pyruvate metabolism. Glucose metabolism ran throughout the entire fermentation process, and the abundances of hexokinase, glucokinase, phosphoglucomutase, and glucose-6-phosphate isomerase always remained at a relatively high level, providing enough precursors for pyruvate metabolism. The pyruvate decarboxylase (EC 4.1.1.1), synthesized by *A. pasteurianus*, and the aldehyde dehydrogenase (NAD+) (EC 1.2.1.3), synthesized by *P. fluorescens* and *S. cerevisiae*, as well as the aldehyde dehydrogenase (NAD(P)+) secreted by *S. cerevisiae*, were responsible for the conversion of the accumulated pyruvate and acetate into acetaldehyde. The abundance of aldehyde dehydrogenase (NAD+) remained at a relatively high level during fermentation, supplying sufficient acetaldehyde for subsequent alcohol metabolism. Throughout the entire fermentation process, acetaldehyde was reduced to alcohol via the alcohol dehydrogenase (EC 1.1.1.1 and EC 1.1.1.2) and alcohol dehydrogenase (cytochrome c) (EC 1.1.2.8) synthesized by functional microbes including *L*. *fermentum*, *L*. *plantarum*, *L*. *uvarum*, *P*. *fluorescens*, *S*. *cerevisiae*, *Weissella confusa*, and *A*. *pasteurianus*. The abundance of alcohol dehydrogenase (EC 1.1.1.1) remained at a high level, especially in the middle and later stages of fermentation, which could ensure the generation of alcohol. The alcohol consumption rate, such as alcohol acidification, esterification, and synthesis to EC, and the generation rate tended to be consistent, which may be the reasons why the alcohol content remained stable in the later stages. The starch and sucrose metabolism, glycolysis/gluconeogenesis, pyruvate metabolism, and alcohol metabolism, along with the fermentation process, provided sufficient alcohol precursor substances for the subsequent synthesis of EC.

The KEGG pathway analysis indicated that dominant precursors of EC formation such as urea and citrulline were mainly synthesized by means of arginine biosynthesis and metabolism. The lactic acid bacteria, *Pseudomonas* and *S*. *cerevisiae* were the primary potential microbes participating in arginine biosynthesis and metabolism. The abundances of glutamine synthetase (EC 6.3.1.2), ornithine carbamoyltransferase (EC 2.1.3.3), ADI (EC 3.5.3.6), acetylornithine deacetylase (EC 3.5.1.16), and carbamate kinase (EC 2.7.2.2), respectively, concerning the formation of glutamate, citrulline, and carbamoyl-P and the degradation of arginine were relatively high. And they were significantly different among the fermented grains under the four fermentation patterns. In the middle and later stages of fermentation, the abundances of ornithine carbamoyltransferase, carbamate kinase, and ADI, related to the synthesis of citrulline and carbamoyl-P and the degradation of arginine, in the fermented grains of protocols A, C, and D were relatively high. Meanwhile, the abundances of enzymes correlated with the decomposition of citrulline and urea, including deargininosuccinate synthase (EC 6.3.4.5), urea carboxylase (EC 6.3.4.6), and urease subunit beta (EC 3.5.1.5), were relatively low during SAB fermentation. This may result in the accumulation of citrulline and urea during SAB fermentation, which is consistent with the results from the analysis of the citrulline and urea contents.

As discussed previously, changes in the RA of all microbes were bounded by about 20 days, and there may be some differences in the metabolic pathways contributed to by microbes before and after this boundary. Therefore, the discrepancies in the metabolic pathways before and after 20 days of fermentation were analyzed (Figure 7). The results demonstrated that the abundances of enzymes degrading urea and synthesizing arginine were relatively high before 20 days of fermentation. As the fermentation proceeded, the abundances of ornithine carbamoyltransferase, carbamate kinase, and ADI, related to the synthesis of citrulline and carbamoyl-P and the degradation of arginine increased, while those of urea carboxylase, allophanate hydrolase, and urease subunit beta, correlated with the degradation of urea, decreased. This may lead to the accumulation of citrulline, carbamoyl-P, and urea during SAB fermentation. High amounts of citrulline, carbamoyl-P, and urea reacting with alcohol may induce an increase in the EC content.

Overall, the HJJQ operation, especially that in protocol C, may be beneficial for increasing the abundances of alpha-amylase, alpha-glucosidase, aldose 1-epimerase, glucokinase, phosphoglucomutase, and glucose-6-phosphate isomerase and facilitated starch’s degradation into dextrin, maltose, and glucose. The glucose was further metabolized into D-fructose-6P and subsequently entered pyruvate metabolism. Then, the synthesized pyruvate was converted into acetaldehyde due to the high abundances of pyruvate decarboxylase, 2-oxoglutarate/2-oxoacid ferredoxin oxidoreductase subunit alpha, and aldehyde dehydrogenase (NAD+) secreted by *A. pasteurianus*, *Staphylococcus xylosus*, and *P. fluorescens*. And acetaldehyde was ultimately metabolized into alcohol via alcohol dehydrogenase. Meanwhile, protocol C was favorable for the functional microbes synthesizing the functional enzymes for arginine metabolism, including arginase, urease subunit beta, glutamine synthetase, ornithine carbamoyltransferase, carbamate kinase, ADI, and argininosuccinate lyase. These enzymes could be used to synthesize EC precursors including citrulline, urea, and carbamoyl-P and increase the EC contents in fermented grains during the later stages of SAB fermentation. This was consistent with the analysis results of the EC contents.

### 3.6. Changes in EC Precursors and EC Contents During SAB Distillation

During SAB distillation, 100 mL of base SAB was sampled per minute until the distillation finished in order to investigate EC’s formation mechanism. The alcohol, HCN, urea, and citrulline contents in base SAB were determined. Due to the high boiling points of urea (about 196.60 °C) and citrulline (about 386.70 °C), they cannot be condensed into base SAB during distillation, and most of these compounds thus remain in the residue, which is consistent with the analysis results for the urea and citrulline contents in the fermented grains. As a result, urea and citrulline were not found in base SAB (or their concentrations were below the detection limits), revealing that they were not the main EC precursors in base SAB, as is consistent with previous reports [12,45,46]. The alcohol, HCN, and EC contents of base SAB during distillation are depicted in Figure S7. The alcohol content continuously decreased as the distillation proceeded. The boiling point of HCN (<30 °C) is lower than that of alcohol (about 78.3 °C), hence changes in the HCN content of base SAB were similar to those for alcohol. The changes in EC content during the distillation process were similar to those for the HCN and alcohol contents. At the initial stage of distillation, the higher the level of HCN and alcohol, the greater the quantity of EC produced, as is consistent with a previous report [12]. Their contents in the head base SAB were far higher than in the heart and tail base SAB, as was consistent with the results reported by Ding et al. [47]. Hence, individually collecting the head base SAB during SAB distillation would be beneficial for reducing the EC content in base SAB. When the HCN and alcohol contents reached about 250 μg/L and 35%, respectively, the EC contents in base SAB tended to be stable. These results suggested that the reaction between HCN and alcohol may have a chemical equilibrium constant and that HCN may not be completely converted into EC during distillation.

**Figure 9:**
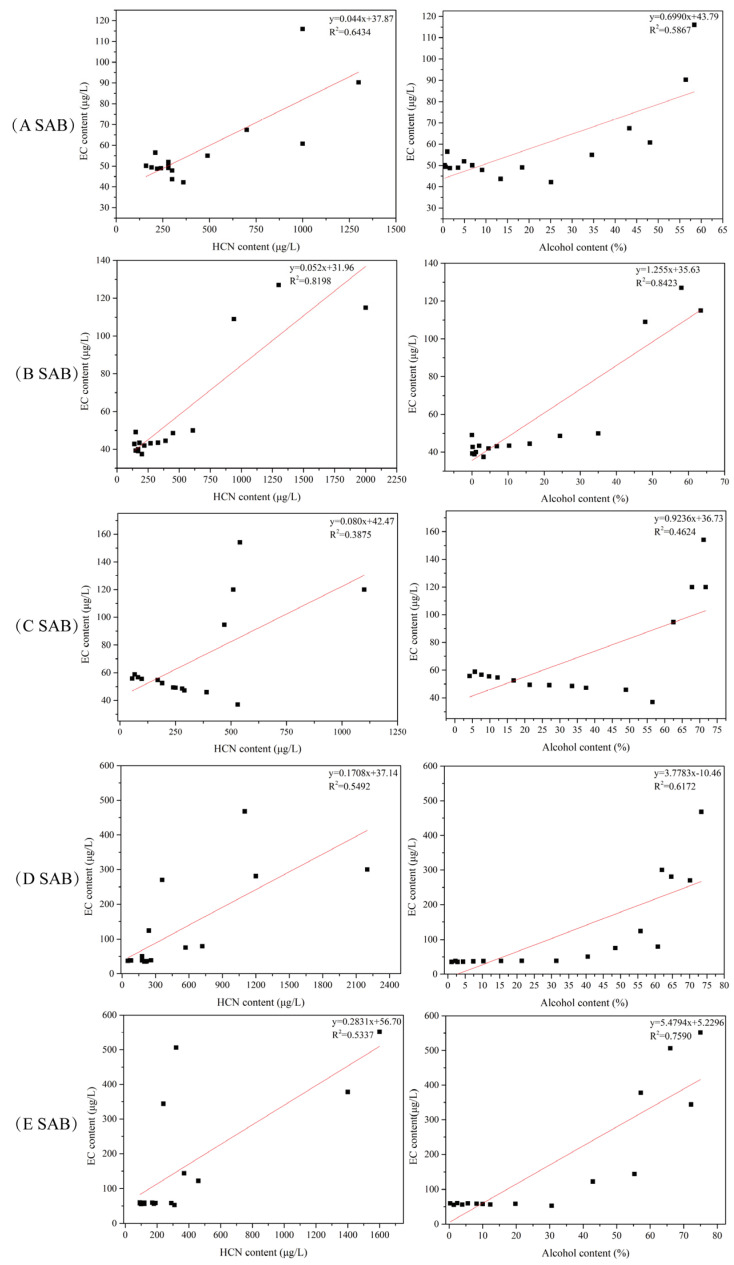
Relationships between EC and EC precursors during SAB distillation. The squares represent the base SAB sampled during distillation. And the red lines represent the linear relationships between EC and EC precursors.

Similar to the investigation on EC formation during SAB fermentation, linear regression and bi-variate correlation analysis were conducted based on the mean values for the alcohol, HCN, and EC contents to analyze the relationships between EC, alcohol, and HCN during distillation (Figure 9). The results demonstrated that the linear relationships between the contents of EC and HCN (R^2^: 0.3875 to 0.8198) and alcohol (R^2^: 0.4642 to 0.8423) were good; the confidence level was at 0.05. It was also indicated that HCN and alcohol were the dominant EC precursors in base SAB during distillation, as was consistent with the results reported by Zhang and Di et al. [12,48].

### 3.7. EC Precursor and EC Contents of Base SAB Fermented Using Different Fermentation Patterns

**Figure 10:**
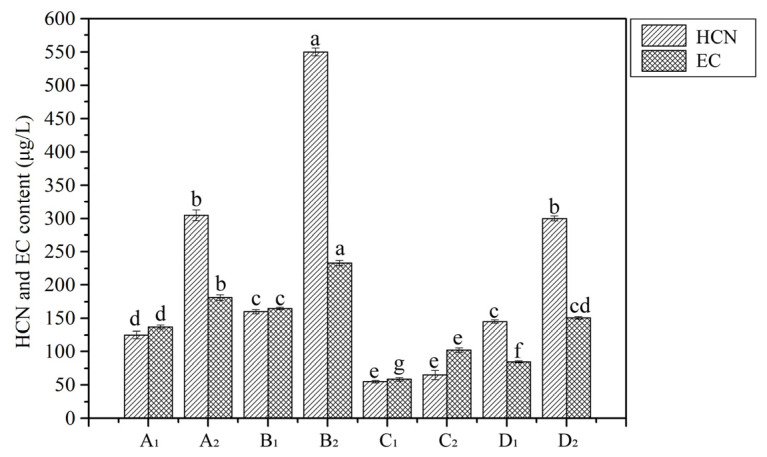
HCN and EC contents of basic SAB. A_1_, B_1_, C_1_, and D_1_ (upper layer) or A_2_, B_2_, C_2_, and D_2_ (lower layer) were the base SAB originating from the upper layer and lower layer of the fermented grains of protocols A, B, C, and D, respectively. Lowercase letters a to g obtained by ANNOVA analysis indicate the significant differences (*p* < 0.05).

As depicted in Figure 3d, the urea content in the final fermented grain obtained by means of protocol A (7.93 ± 0.22 mg/kg) was significantly lower than that obtained by means of protocol B (19.37 ± 0.04 mg/kg), while that obtained by means of protocol C (16.25 ± 0.08 mg/kg) was slightly lower than that obtained by means of protocol D (18.68 ± 0.12 mg/kg). The EC content in the final fermented grain obtained by means of protocol C (192.42 ± 2.30 μg/kg) was the highest, followed by D (171.44 ± 4.50 μg/kg) and A (163.83 ± 1.18 μg/kg), while that obtained by means of protocol B (66.82 ± 2.52 μg/kg) was the lowest. The HCN content in the final fermented grain obtained by means of protocol B (230.67 ± 9.10 μg/kg) was the highest, followed by D (132.59 ± 7.16 μg/kg), while those obtained by means of protocols A (73 ± 4.68 μg/kg) and C (68.09 ± 3.17 μg/kg) were the lowest. After the final fermented grains were distilled, the HCN and EC contents in base SAB were determined and are shown in Figure 10. Compared with the HCN and EC contents in base SAB distilled from the fermented grains of protocol B, their contents in base SAB distilled from the same layer of the fermented grains of protocol A were lower. Similar trends were also observed in the HCN and EC contents of base SAB distilled from the fermented grains of protocols D and C. The results suggest that the HCN and EC contents in base SAB originating from the fermented grains with a high HCN content were relatively high, while the urea and EC contents in the fermented grains have little effect on the EC content in base SAB. Owing to the high boiling points of urea and EC, only small amounts of the urea and EC produced during the fermentation process could be evaporated into the final distillate [12,38]. Therefore, the EC in base SAB was mostly formed during the distillation process, and the EC formed during distillation was an important part of the EC in the base SAB. This was consistent with the results reported previously [12]. The residual urea and EC in *Zaopei* were transferred into the next round of fermentation. Overall, the HCN contents in fermented grains had a remarkable influence on the EC content in base SAB. The HJJQ operation, especially in protocol C, was beneficial for reducing the HCN content and increasing the EC content in the fermented grains and further reducing the EC formation from HCN during distillation.

### 3.8. Sensory Properties of Base SAB Fermented Using Different Fermentation Patterns

The mean scores of nine sensory evaluation dimensions, namely, transparency, aroma, softness, sweetness, fullness, pure taste, aftertaste, stale taste, and features, are shown in Figure S8. The results suggest that all evaluation dimensions exhibited significant differences among the base SAB samples. The sensory scores of the base SAB obtained from the sub layer of fermented grains were higher than those of the base SAB obtained from the upper layer of fermented grains. The sensory scores of A_1_, A_2_, C_1_, and C_2_ were the highest and showed high transparency, an intense aroma, smooth softness, mellow and sweetness, moderate fullness, a moderate pure taste, a long-lasting aftertaste, an ordinary stale taste, and typical features, followed by B_2_ and B_1_, while the scores of D_2_ and D_1_ were the lowest. During SAB fermentation, the HJJQ operation adopted in protocols A and C was favorable for increasing the alcohol content and promoting the esterification reaction between alcohol and acids. Therefore, the quality of the base SAB originating from the fermented grains produced using protocols A and C was relatively higher than that of the others.

## 4. Conclusions

Changes in the physiochemical indices, EC precursors, EC contents, and the microbial community of fermented grains under different fermentation patterns during SAB fermentation, as well as changes in EC precursors and EC contents during distillation were investigated and discussed. The results suggested that, in the primary fermentation period, the higher the amount of glutinous sorghum added, the higher the fermentation temperature and starch and reducing sugar contents detected in the fermented grains. The HJJQ operation could significantly reduce the acidity of the fermented grains and increase the alcohol content in fermented grains but had little effect on the other physicochemical indices during fermentation. The arginine, citrulline, and EC contents in the final fermented grains of protocols C and D were higher than those of protocols A and B. The urea and HCN contents in the final fermented grains of protocols B and D were higher than those of protocols A and B. HJJQ could facilitate the reaction between alcohol and EC precursors, decreasing EC precursor content and increasing EC content as a result. Mother *Zaopei* was also a dominant source of EC precursors and EC. Correlation analysis indicated that alcohol, citrulline, urea, and HCN were the predominant EC precursors during SAB fermentation. KEGG pathway analysis demonstrated that alcohol, urea, and citrulline were mainly synthesized through alcohol and arginine metabolism. The HJJQ operation facilitates the lactic acid bacteria, *Pseudomonas*, *Acetobacter*, and *Staphylococcus* to form enzymes involved in the synthesis of alcohol, urea, and citrulline. The changes in alcohol, HCN, and EC contents were similar to each other, and the former two were the primary EC precursors during distillation. The urea and EC contents in the fermented grains had little effect on the EC content in the base SAB, and the EC in the base SAB was mostly formed from HCN during distillation. Overall, the HJJQ operation, especially in protocol C, could significantly reduce the EC content in base *Baijiu*, making the base *Baijiu* obtained of better quality. The HJJQ operation presented in this study may be an alternative and effective way to eliminate the EC content in base *Baijiu*. Expanding the application of this technology to *Baijiu* manufactures would reduce the EC content to a lower level, ensuring the safety of *Baijiu* and further protecting consumer health.

## Data Availability

The original contributions presented in this study are included in the article/Supplementary Materials. Further inquiries can be directed to the corresponding author.

## Supplementary Materials

The following supporting information can be downloaded at: https://www.mdpi.com/article/10.3390/foods13233743/s1, Figure S1. KEGG annotation results based on genome database of microbes presented in fermented grains during SAB fermentation. Figure S2. Changes in the functional contributions of microbes to coding genes of key enzymes in starch metabolic pathways. Figure S3. Changes in the functional contributions of microbes to coding genes of key enzymes in glucose metabolic pathways. Figure S4. Changes in the functional contributions of microbes to coding genes of key enzymes in pyruvate metabolic pathways. Figure S5. Changes in the functional contributions of microbes to coding genes of key enzymes in alcohol metabolic pathways. Figure S6. Changes in the functional contributions of microbes to coding genes of key enzymes in arginine biosynthesis and metabolic pathways. Figure S7. Changes in alcohol, HCN and EC contents of base SAB during distillation. Figure S8. Sensory properties of base SAB fermented by different fermentation patterns; Table S1. Evaluation dimensions and score range; Table S2. Changes in elative abundance of functional microbes during SAB fermentation; Table S3. Changes in the coding gene abundance of key enzymes involved in the metabolic pathways of EC precursors during SAB fermentation; Table S4. Changes in the functional contributions of microbes to coding genes of key enzymes involved in the metabolic pathways of EC precursors during SAB fermentation.

## Abbreviations

The following abbreviations are used in this manuscript:

SAB	Strong-aroma *Baijiu*
HJJQ	Huijiu jiaqu
EC	Ethyl carbamate
HCN	Cyanide
FFB	Fen-flavor *Baijiu*
MFB	Maotai-flavor *Baijiu*
MOE	Margin of exposure
HPLC	High-performance liquid chromatography
ADI	Arginine deiminase
