# Supplementary material for: Decoding the Formation and Elimination Mechanism of Ethyl Carbamate in Strong-Aroma Baijiu
Source: Foods. 2024 Nov 22;13(23):3743. doi: 10.3390/foods13233743 (PMC11640385; doi:10.3390/foods13233743)
Supplement: Supplementary file 1 [file foods-13-03743-s001.zip › Figure captions.pdf]

Figure captions

Figure S1 KEGG annotation results based on genome database of microbes presented in fermented grains during SAB fermentation.

Figure S2 Changes in the functional contributions of microbes to coding genes of key enzymes in starch metabolic pathways.

Figure S3 Changes in the functional contributions of microbes to coding genes of key enzymes in glucose metabolic pathways.

Figure S4 Changes in the functional contributions of microbes to coding genes of key enzymes in pyruvate metabolic pathways.

Figure S5 Changes in the functional contributions of microbes to coding genes of key enzymes in alcohol metabolic pathways.

Figure S6 Changes in the functional contributions of microbes to coding genes of key enzymes in arginine biosynthesis and metabolic pathways.

Figure S7 Changes in alcohol, HCN and EC contents of base SAB during distillation.

Figure S8 Sensory properties of base SAB fermented by different fermentation patterns.

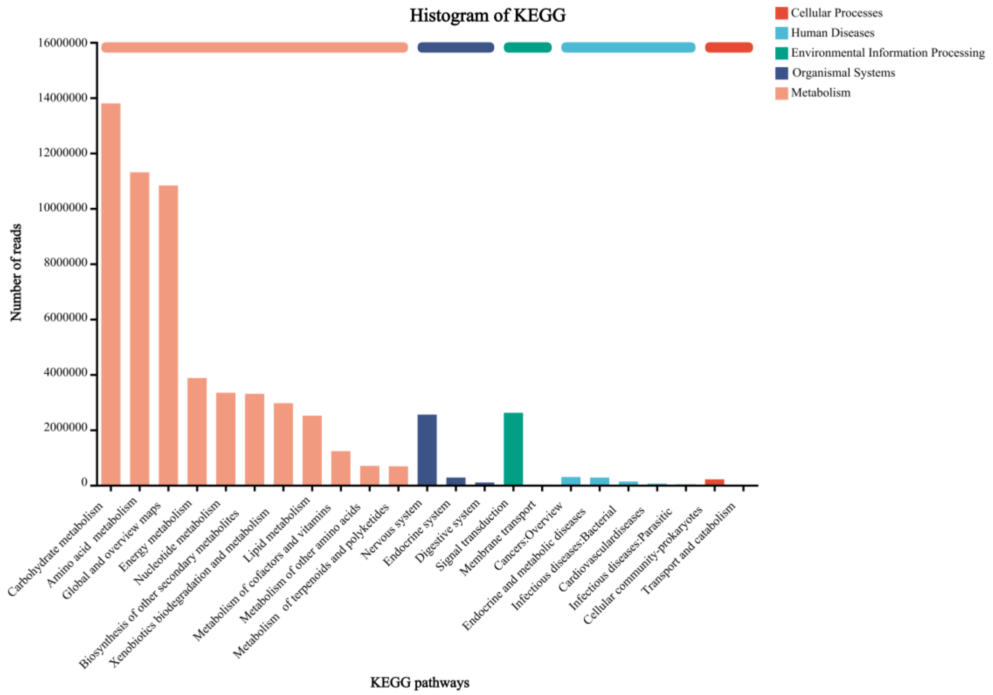

Histogram of KEGG
Cellular Processes
Human Diseases
Environmental Information Processing
Organismal Systems
Metabolism
Number of reads
16000000
14000000
12000000
10000000
8000000
6000000
4000000
2000000
0
Carbohydrate metabolism
Amino acid metabolism
Global and overview maps
Energy metabolism
Nucleotide metabolism
Biosynthesis of other secondary metabolites
Xenobiotics biodegradation and metabolism
Lipid metabolism
Metabolism of cofactors and vitamins
Metabolism of other amino acids
Metabolism of terpenoids and polyketides
Nervous system
Endocrine system
Digestive system
Signal transduction
Membrane transport
Cancers:Overview
Endocrine and metabolic diseases
Infectious diseases:Bacterial
Cardiovasculardiseases
Infectious diseases:Parasitic
Cellular community-prokaryotes
Transport and catabolism
KEGG pathways

Fig. S1

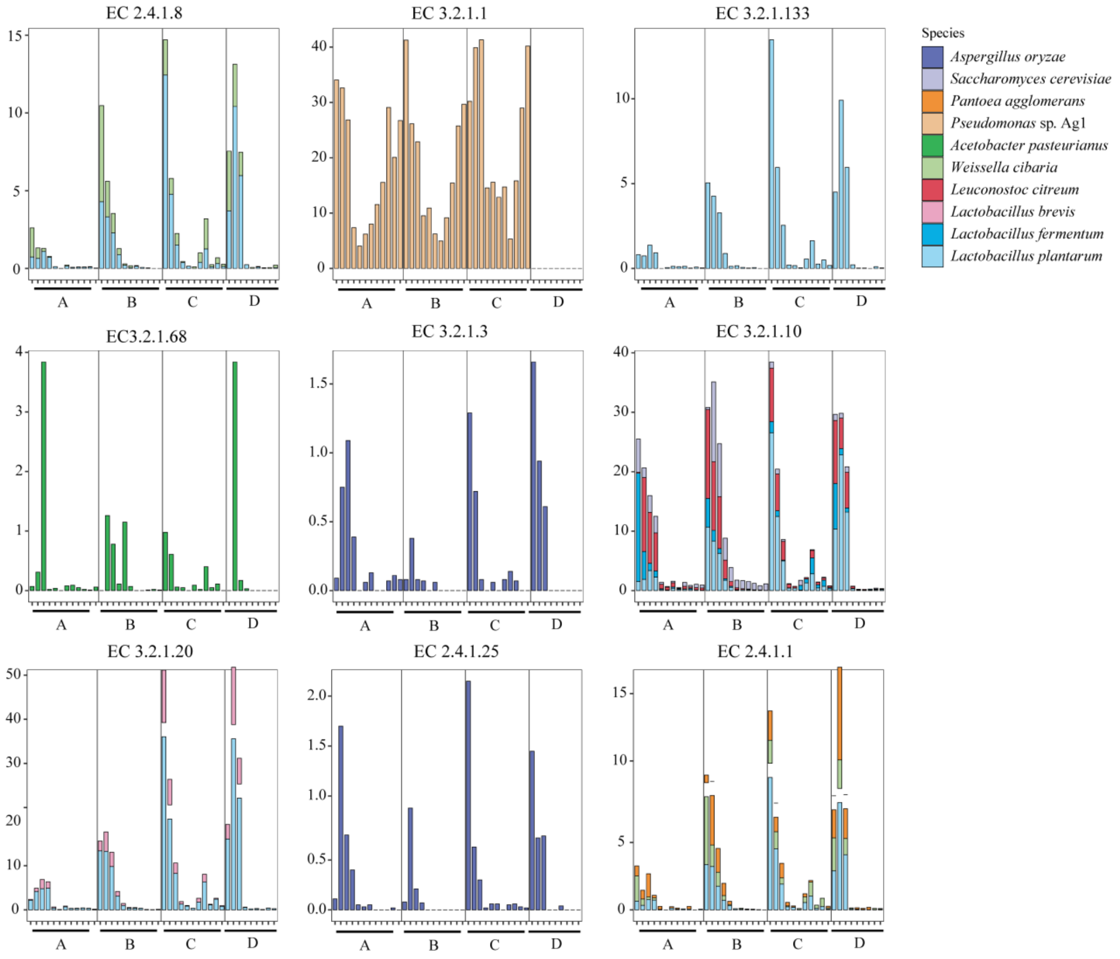
EC 2.4.1.8
EC 3.2.1.1
EC 3.2.1.133
EC3.2.1.68
EC 3.2.1.3
EC 3.2.1.10
EC 3.2.1.20
EC 2.4.1.25
EC 2.4.1.1
A
B
C
D
Species
Aspergillus oryzae
Saccharomyces cerevisiae
Pantoea agglomerans
Pseudomonas sp. Ag1
Acetobacter pasteurianus
Weissella cibaria
Leuconostoc citreum
Lactobacillus brevis
Lactobacillus fermentum
Lactobacillus plantarum

Fig. S2

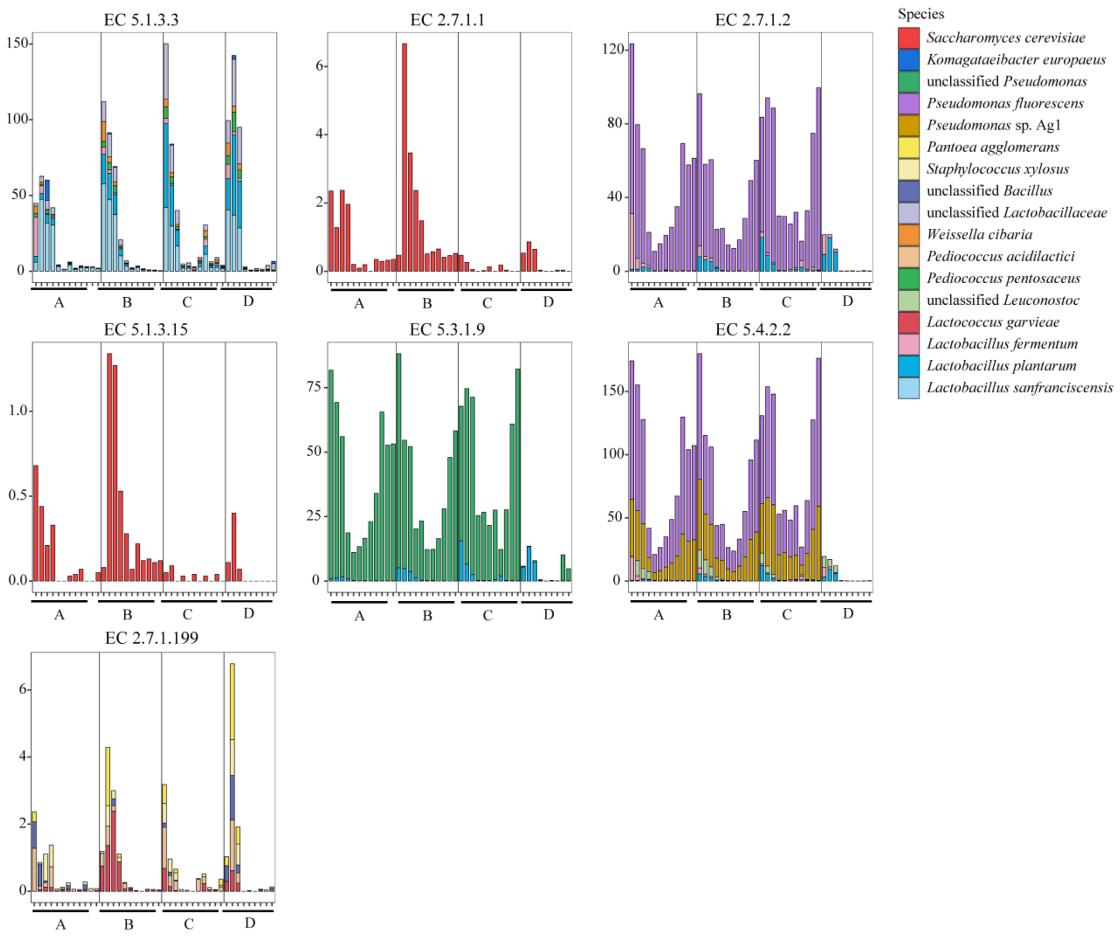
EC 5.1.3.3
EC 2.7.1.1
EC 2.7.1.2
EC 5.1.3.15
EC 5.3.1.9
EC 5.4.2.2
EC 2.7.1.199
A
B
C
D
Species
Saccharomyces cerevisiae
Komagataeibacter europaeus
unclassified Pseudomonas
Pseudomonas fluorescens
Pseudomonas sp. Ag1
Pantoea agglomerans
Staphylococcus xylosus
unclassified Bacillus
unclassified Lactobacillaceae
Weissella cibaria
Pediococcus acidilactici
Pediococcus pentosaceus
unclassified Leuconostoc
Lactococcus garvieae
Lactobacillus fermentum
Lactobacillus plantarum
Lactobacillus sanfranciscensis

Fig. S3

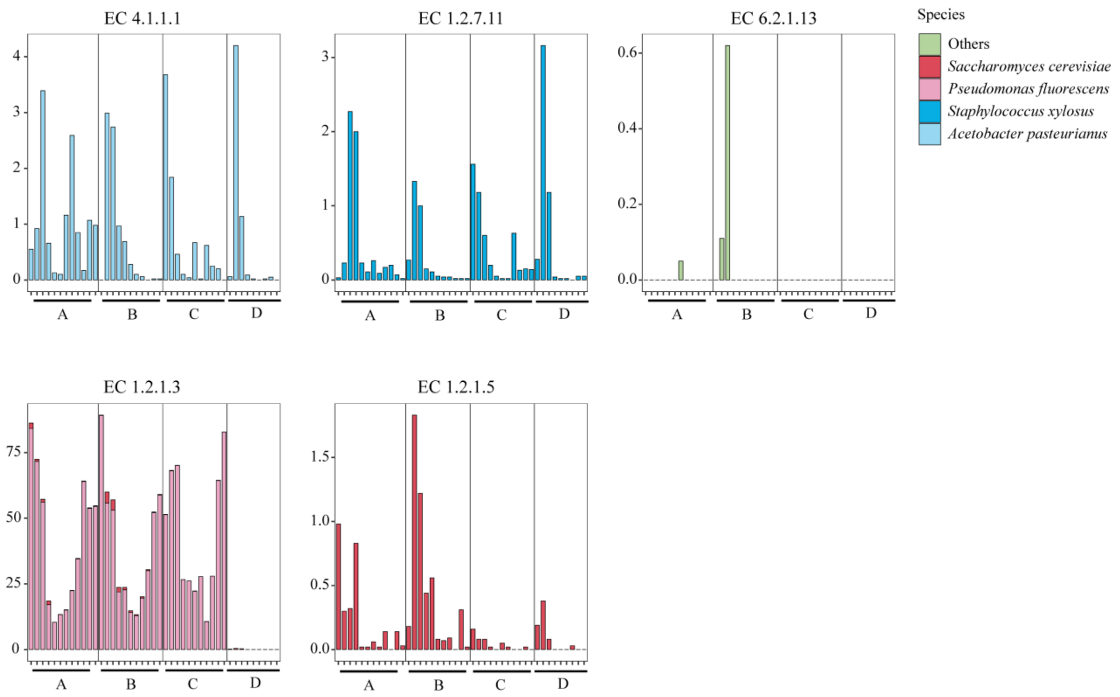

EC 4.1.1.1
EC 1.2.7.11
EC 6.2.1.13
EC 1.2.1.3
EC 1.2.1.5
Species
Others
Saccharomyces cerevisiae
Pseudomonas fluorescens
Staphylococcus xylosus
Acetobacter pasteurianus
A
B
C
D

Fig. S4

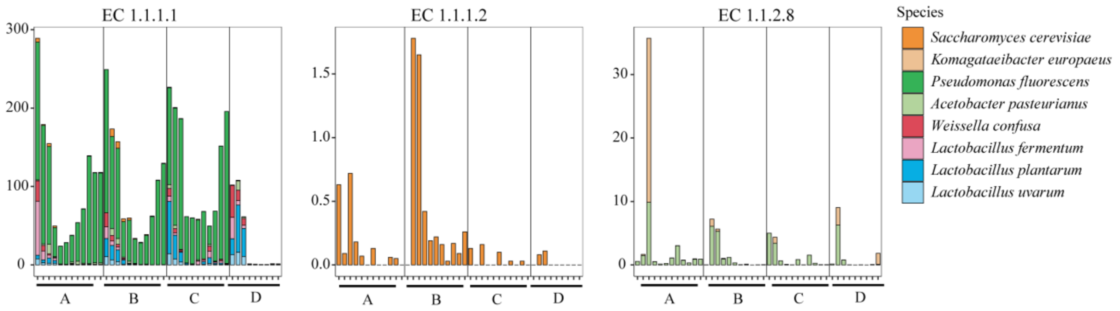
EC 1.1.1.1
EC 1.1.1.2
EC 1.1.2.8
300
200
100
0
1.5
1.0
0.5
0.0
30
20
10
A
B
C
D
Species
Saccharomyces cerevisiae
Komagataeibacter europaeus
Pseudomonas fluorescens
Acetobacter pasteurianus
Weissella confusa
Lactobacillus fermentum
Lactobacillus plantarum
Lactobacillus uvarum

Fig. S5

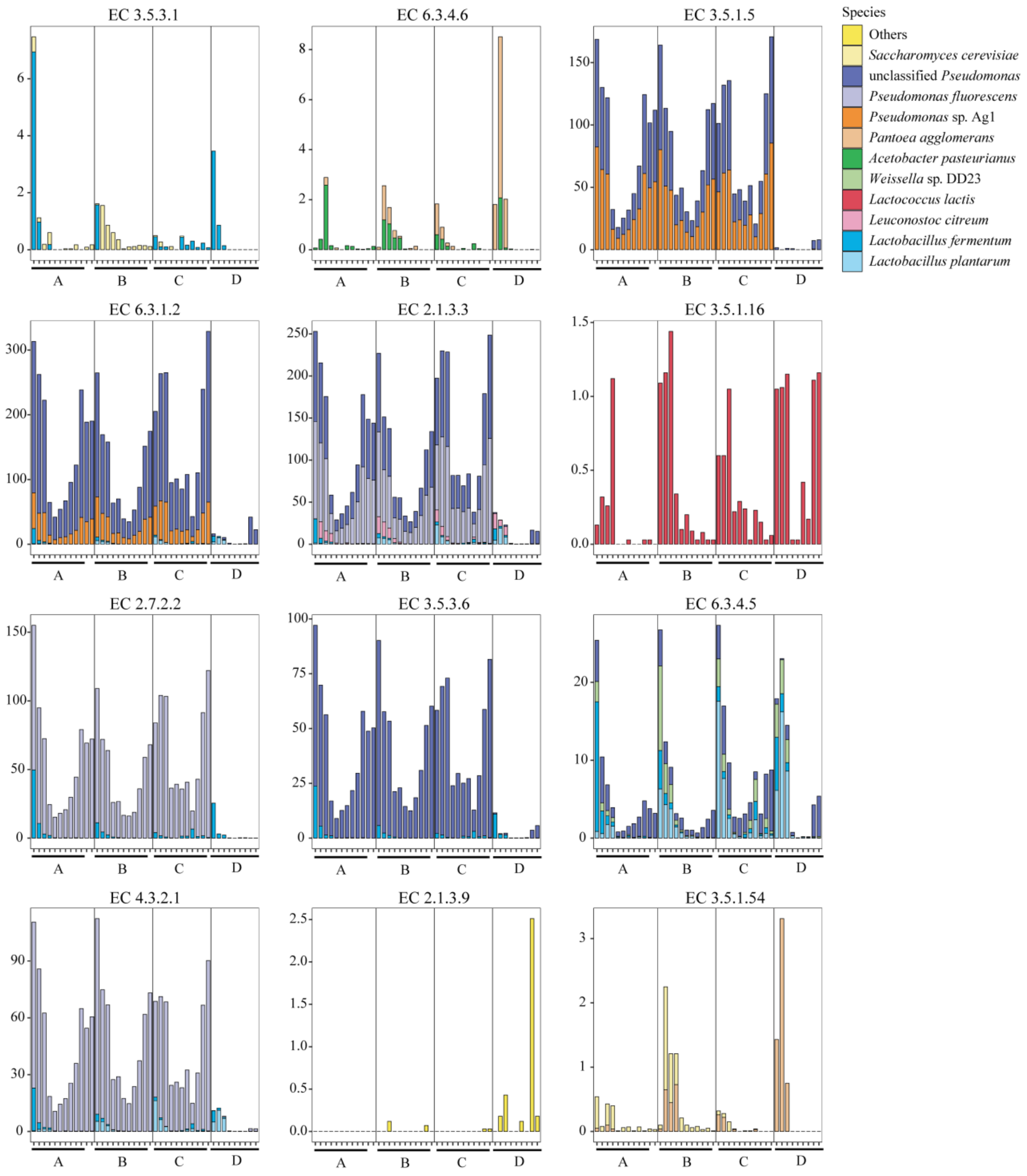
EC 3.5.3.1
EC 6.3.4.6
EC 3.5.1.5
EC 6.3.1.2
EC 2.1.3.3
EC 3.5.1.16
EC 2.7.2.2
EC 3.5.3.6
EC 6.3.4.5
EC 4.3.2.1
EC 2.1.3.9
EC 3.5.1.54
A
B
C
D
Species
Others
Saccharomyces cerevisiae
unclassified Pseudomonas
Pseudomonas fluorescens
Pseudomonas sp. Ag1
Pantoea agglomerans
Acetobacter pasteurianus
Weissella sp. DD23
Lactococcus lactis
Leuconostoc citreum
Lactobacillus fermentum
Lactobacillus plantarum

Fig. S6

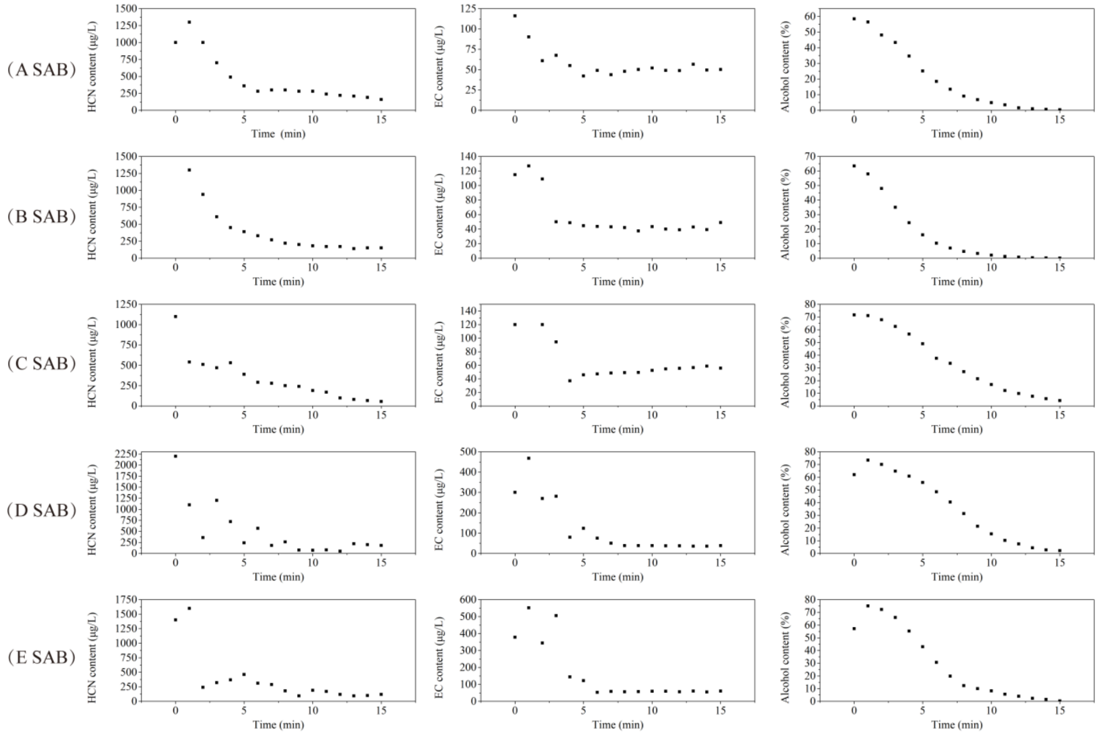
(A SAB)
(B SAB)
(C SAB)
(D SAB)
(E SAB)
HCN content (μg/L)
EC content (μg/L)
Alcohol content (%)
Time (min)

Fig. S7

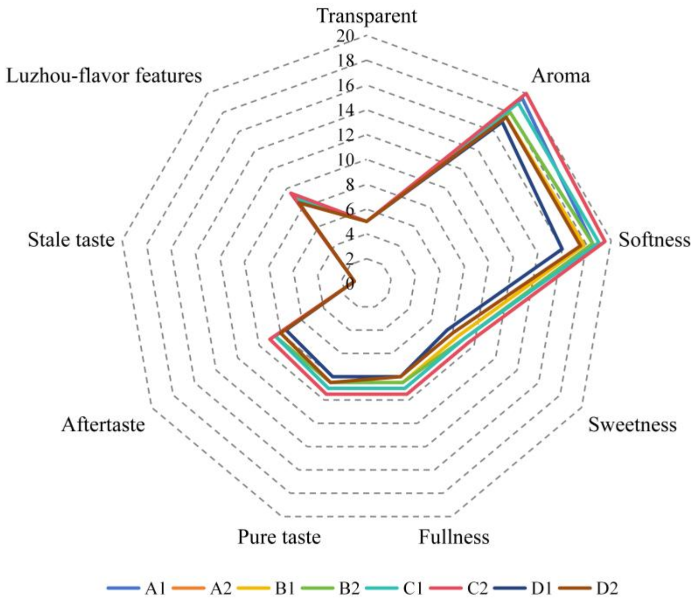
Transparent
Aroma
Softness
Sweetness
Fullness
Pure taste
Aftertaste
Stale taste
Luzhou-flavor features
20
18
16
14
12
10
8
6
4
2
0
A1
A2
B1
B2
C1
C2
D1
D2

Fig. S8
