# Supplementary material for: Decoding the Formation and Elimination Mechanism of Ethyl Carbamate in Strong-Aroma Baijiu
Source: Foods. 2024 Nov 22;13(23):3743. doi: 10.3390/foods13233743 (PMC11640385; doi:10.3390/foods13233743)
Supplement: Supplementary file 1 [file foods-13-03743-s001.zip › Table S1 Sensory Quality Score.pdf]

Table S1 Evaluation dimensions and score range

Sensory properties	Intensity	Score
Transparency	High	5
	Moderate	4~4.5
	Low	2~3.5
Aroma	Very intense	19.5~20
	Intense	18.5~19
	Moderate	17.5~18
	Ordinary	14~17
	Weak	11~13
Softness	Very smooth	19.5~20
	Smooth	18.5~19
	Slightly spicy	17.5~18
	Rough and spicy	16~17
Sweetness	Mellow and sweet	9.5~10
	Sweet	8.5~9
	Slightly sweet	7.5~8
Fullness	Intense	9.5~10
	Moderate	8.5~9
	Ordinary	7.5~8
	Weak	5~6
Pure taste	Intense	9.5~10

	Moderate	8.5~9
	Ordinary	7.5~8
	Weak	5~6
Aftertaste	Longer time	9.5~10
	longtime	8.5~9
	Short time	7.5~8
Stale taste	Intense	4.5~5
	Moderate	3.5~4
	Ordinary	1~3
Luzhou-flavor features	Typical	9.5~10
	Moderately typical	8.5~9
	Less typical	7.5~8
